# Repression of Germline RNAi Pathways in Somatic Cells by Retinoblastoma Pathway Chromatin Complexes

**DOI:** 10.1371/journal.pgen.1002542

**Published:** 2012-03-08

**Authors:** Xiaoyun Wu, Zhen Shi, Mingxue Cui, Min Han, Gary Ruvkun

**Affiliations:** 1 Department of Molecular Biology, Massachusetts General Hospital, Boston, Massachusetts, United States of America; 2 Department of Genetics, Harvard Medical School, Boston, Massachusetts, United States of America; 3 Department of Molecular, Cellular, and Developmental Biology, University of Colorado, Boulder, Colorado, United States of America; 4 Howard Hughes Medical Institute, University of Colorado, Boulder, Colorado, United States of America; Harvard University, United States of America

## Abstract

The retinoblastoma (Rb) tumor suppressor acts with a number of chromatin cofactors in a wide range of species to suppress cell proliferation. The *Caenorhabditis elegans* retinoblastoma gene and many of these cofactors, called synMuv B genes, were identified in genetic screens for cell lineage defects caused by growth factor misexpression. Mutations in many synMuv B genes, including *lin-35/Rb*, also cause somatic misexpression of the germline RNA processing P granules and enhanced RNAi. We show here that multiple small RNA components, including a set of germline-specific Argonaute genes, are misexpressed in the soma of many synMuv B mutant animals, revealing one node for enhanced RNAi. Distinct classes of synMuv B mutants differ in the subcellular architecture of their misexpressed P granules, their profile of misexpressed small RNA and P granule genes, as well as their enhancement of RNAi and the related silencing of transgenes. These differences define three classes of synMuv B genes, representing three chromatin complexes: a LIN-35/Rb-containing DRM core complex, a SUMO-recruited Mec complex, and a synMuv B heterochromatin complex, suggesting that intersecting chromatin pathways regulate the repression of small RNA and P granule genes in the soma and the potency of RNAi. Consistent with this, the DRM complex and the synMuv B heterochromatin complex were genetically additive and displayed distinct antagonistic interactions with the MES-4 histone methyltransferase and the MRG-1 chromodomain protein, two germline chromatin regulators required for the synMuv phenotype and the somatic misexpression of P granule components. Thus intersecting synMuv B chromatin pathways conspire with synMuv B suppressor chromatin factors to regulate the expression of small RNA pathway genes, which enables heightened RNAi response. Regulation of small RNA pathway genes by human retinoblastoma may also underlie its role as a tumor suppressor gene.

## Author Summary

In metazoans, soma and germline have specialized functions that require differential tissue-specific gene expression. In *C. elegans*, explicit chromatin marks deposited by the MES-4 histone methyltransferase and the MRG-1 chromodomain protein allow germline expression of particular suites of target genes. Conversely, the expression of germline-specific genes is repressed in somatic cells by other chromatin regulatory factors, including the retinoblastoma pathway genes. We characterized the distinct profiles of somatic misexpression of normally germline-specific genes in these mutants and mapped out three chromatin complexes that prevent misexpression. We demonstrate that one of the complexes closely counteracts the activity of MES-4 and MRG-1, whereas another complex interacts with additional regulators that are yet to be identified. We show that these intersecting chromatin complexes prevent the upregulation of a suite of germline-specific as well as ubiquitous small RNA pathway genes, which contributes to the enhanced RNAi response in retinoblastoma pathway mutant worms. We suggest that this function of the retinoblastoma pathway chromatin factors to prevent germline-associated gene expression programs in the soma and the upregulation of small RNA pathways may also underlie their role as tumor suppressors.

## Introduction

The tumor suppressor protein Rb (retinoblastoma) is a chromatin factor that functions in transcriptional repression of cell cycle regulatory genes as well as other genes. As a transcriptional repressor, Rb functions in a core complex (dREAM/Muv B) that binds to specific promoters and recruits a crew of repressive chromatin cofactors to inhibit the expression of target genes [1], [2]. Rb-recruited factors include repressive histone methyltransferases (Suv39, Suv420), repressive heterochromatin proteins that bind to methylated histones (HP-1, L3MBT), and the Nucleosome Remodeling and Deacetylase complex (NuRD complex) [3]–[5]. Beyond transcription, Rb also interacts with other chromatin factors (*e.g.*, condensin II) and participates in other chromatin functions such as chromosome condensation and maintaining genome stability [6], [7]. Even though it has been studied as a cell cycle regulator for two decades, the functions of Rb are clearly much broader.

Rb pathway genes have been studied in the nematode *C. elegans* because Rb and many of its interacting proteins identified biochemically in flies and mammals are conserved in *C. elegans* and null alleles in the corresponding *C. elegans* genes cause similar developmental phenotypes [2]. Genes encoding Rb (*lin-35*), the rest of the core DRM/dREAM complex, and Rb-recruited repressive chromatin factors all belong to the class of synMuv B (synthetic multivulva B) genes, mutations in which cause a Muv (Multivulva) phenotype when combined with a mutation in a synMuv A gene. synMuv B genes, along with synMuv A genes, repress the expression of the growth factor gene *lin-3*/EGF in the developing hypodermis [8]. Excess EGF (Epidermal Growth Factor) signaling from the hypodermis in synMuv AB double mutant animals induces multiple vulva precursor cells to adopt the cell division patterns normally specified for only one vulval precursor cell, causing the Muv phenotype. Rb pathway chromatin factors comprise the bulk of the synMuv B genes as revealed by saturation genetic analysis and whole genome RNAi screens in synMuv A mutant strains, from which a few additional synMuv B genes have been identified, some of which also encode probable chromatin factors [9], [10]. The genetic pathways necessary for the Muv phenotype in synMuv AB double mutant worms have been revealed by whole genome RNAi screens for gene inactivations that suppress the Muv phenotype of synMuv AB mutants, which also identified distinct chromatin factors [11]. Therefore, Rb pathway proteins function with particular chromatin factors while antagonizing others to specify the production of the LIN-3/EGF signal from particular cells during vulval development.

Mutations in several synMuv B genes, especially those that encode the Rb core complex also cause a dramatic enhancement of response to dsRNA, that is enhanced RNAi (Eri) [12], [13]. Inactivation of *lin-35/Rb* also causes enhanced transgene silencing, a process that depends on some RNAi factors [12], [13]. Eri and enhanced transgene silencing are also caused by mutations in distinct RNAi regulatory factors, for example, the genes *eri-1* to *eri-9*, that do not interact with synMuv A mutations to induce a Muv phenotype [14], [15]. *lin-35/Rb* likely inhibits RNAi using a distinct mechanism from these other *eri* genes because null alleles in *lin-35* and *eri* genes are genetically additive, have different genetic requirements for canonical RNAi factors, and display specificity in gene inactivation tests involving distinct dsRNAs [13]. One potential mechanism of the enhanced RNAi response in synMuv B mutants is the somatic misexpression of germline-specific genes observed in these animals, given that many RNAi factors are preferentially expressed in the *C elegans* germline which is also more proficient at RNAi [13].

Many synMuv B mutants misexpress germline-specific P granules in their somatic tissue [13], [16], [17]. Homologous to nuage and polar granules of insects and mammals, P granules mark the germline of *C. elegans* from the very first cell divisions and are essential to the function and maintenance of the germline [18], [19]. These perinuclear RNP granules harbor processing and binding proteins for mRNAs as well as endogenous small RNAs, and are thought to be the site of nascent mRNA export and endogenous small RNA biogenesis and storage [20]–[22]. The somatic misexpression of P granule components was first observed in *mep-1* and *let-418* mutants which were found to also function in the synMuv B pathway [17]. Unlike null mutants of most synMuv B genes, which are viable, null mutations of *mep-1* or *let-418* cause L1 arrest or sterility, suggesting that the expression of germline P granules in somatic cells can have severe developmental consequences or that these genes also regulate other essential functions in contrast to *lin-35/Rb*. The somatic misexpression of germline P granules as well as the developmental arrest phenotypes of *mep-1* or *let-418* are fully suppressed by inactivation of three synMuv B suppressor genes, which encode germline chromatin factors (*mes-4*, *mrg-1*, *isw-1*), pointing to antagonistic chromatin factors in regulating the expression of germline genes in the soma [11], [23], [24]. It was proposed that the NuRD complex, containing LET-418, prevents somatic misexpression through chromatin modification or remodeling and antagonizing the function of germline specific chromatin factors [17]. Later, the misexpression of P granules in somatic cells was shown in several other synMuv B mutants, including *lin-35/Rb*, indicating a broader involvement of synMuv B genes in this process [13]. While these synMuv B mutants are viable, all of them display high temperature larval arrest, again suggesting severe developmental consequences from such misexpression of normally germline-specific genes [16]. Loss of the germline chromatin factors *mes-4, mrg-1 and isw-1*, which suppresses the somatic misexpression and high temperature larval arrest in these other synMuv B mutants, was also shown to suppress the enhanced transgene silencing and vulval specification phenotypes, suggesting that the somatic misexpression of germline components contributes to the enhanced RNAi and the vulval cell fate change in these animals [13], [16], [23]–[25]. The inappropriate expression of germline genes including small RNA pathway components could also contribute to the oncogenicity of loss of Rb in mammalian cells.

Both the enhanced RNAi and the expression of germline-specific genes in soma uniquely reflect the function of the synMuv B pathway, as they are not present in mutants of other synMuv classes. However, not all synMuv B mutants are Eri or show PGL-1 misexpression, suggesting functional specialization. Moreover, only some of the Muv suppressing chromatin factors affect RNAi and only three (*mes-4, mrg-1, isw-1*) suppress PGL-1 misexpression [11]. Thus, the genetic pathways underlying Eri and the expression of germline genes in somatic cells remain to be further investigated.

Here we use response to RNAi, subcellular analysis of somatic P granules, and characterization of somatic misexpression of germline-specific genes to reveal that synMuv B genes constitute distinct intersecting axes for the repression of germline genes in somatic cells. We find that these chromatin factors regulate the expression of partially overlapping sets of germline target genes, including genes that encode annotated small RNA cofactors such as Argonaute proteins, and P granule components such as helicases and RNA binding proteins. The three synMuv B chromatin complexes we find are the LIN-35/Rb-containing core complex (DRM), the SUMO-mediated Mec complex (rather than the previously suspected NuRD complex), and a synMuv B heterochromatin complex, representing distinct classes of synMuv B mutants. We show that the DRM and synMuv B heterochromatin complexes each have distinct requirements for MES-4 function, suggesting different placements in the genetic pathway. We present a model of how synMuv B complexes collectively inhibit RNAi and prevent germline gene expression in the soma.

## Results

### Three synMuv B functional classes based on molecular and biochemical association

The majority of synMuv B genes revealed by saturation genetic and RNAi screens encode chromatin factors that belong to three functional classes: the DRM complex, a predicted NuRD complex, and several heterochromatin proteins (Figure S1). The worm DRM complex (dREAM/MMB in flies and DREAM in mammals) consists of LIN-35/Rb, the Rb binding protein LIN-53/RbBP4, the transcription factor dimer EFL-1/E2F4-DPL-1/DP1, and homologues of Myb-interacting proteins LIN-9/Mip130, LIN-54/Mip120 and LIN-37/Mip40 [3], [26]–[28]. LIN-53/RbBP4 is a shared component with the mammalian NuRD complex. The *C. elegans* NuRD complex has not been biochemically defined, but homologues of mammalian NuRD have emerged from genetic analyses. For example, genes encoding the histone deacetylase HDA-1/Rpd3 and the ATP-dependent chromatin remodeling enzyme LET-418/Mi-2 act in synMuv B pathways [17], [29], [30]. Although MEP-1 is not part of the mammalian NuRD due to the lack of an obvious homologue, it physically and functionally interacts with LET-418 in worms and flies [17]. synMuv B heterochromatin proteins include histone methyltransferases MET-1 and MET-2, methyl histone binding proteins HPL-2/HP1 and LIN-61/L3MBT, and the multiple zinc finger protein LIN-13, which mediates HPL-2 localization via physical interaction [31]–[34]. The molecular functions of the remaining synMuv B proteins are less known. These include LIN-15B, LIN-36, TAM-1, RPB-11, E01A2.4, GEI-4 and LIN-65 [9], [10], [35]–[38].

### Phenotypic classification of synMuv B genes

**Figure 1:**
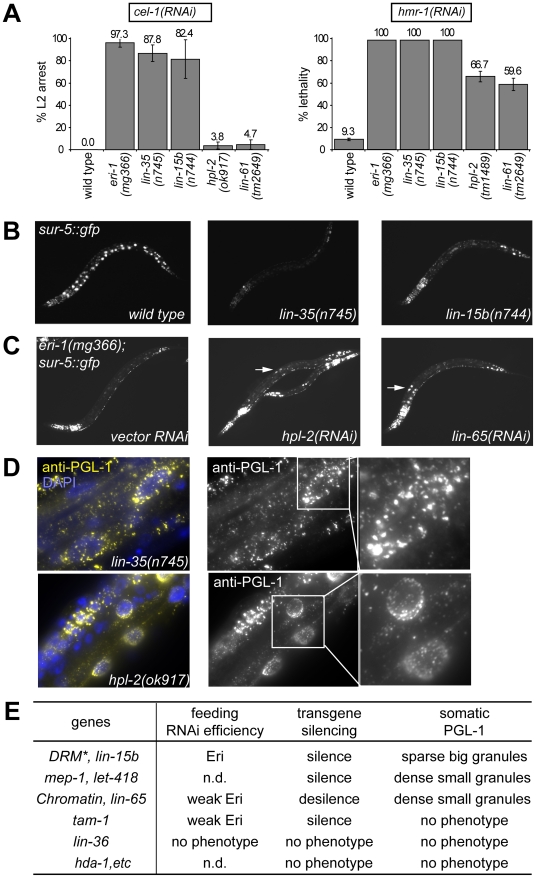
Distinct synMuv B mutant classes affect RNAi and PGL-1 misexpression differently. (A) RNA interference responses to gene inactivations that are poor in wild type but enhanced in synMuv B mutants. L1 animals of the indicated genotype were fed *E. coli* expressing dsRNA for the listed tester genes and phenotypes were scored in the next generation (F1). % L2 arrest represents the percentage of F1 animals arresting at L2 stage. % lethality represents the percent reduction of F1 progeny from animals treated with RNAi compared to control. (B) Mutants of the DRM complex displayed enhanced transgene silencing. L3 animals with indicated genotypes carrying a *sur-5::gfp* transgene were imaged for *gfp* expression. (C) Inactivation of synMuv B heterochromatin class genes causes transgene desilencing. *eri-1(mg366); sur-5::gfp* animals treated with the indicated RNAi were imaged for *gfp* expression at the L3 stage. Arrows point to intestinal nuclei with brighter GFP expression indicating transgene desilencing. (D) Different PGL-1 misexpression phenotypes displayed by representative mutants. L4 or young adult animals were stained with anti-PGL-1 antibody K76 (yellow) and DAPI (blue). Arrows point to the intestinal nuclei whose PGL-1 staining patterns are shown in the insets. (E) Summary of the classification of synMuv B genes based on phenotypes. *: excluding *efl-1*. n.d.: not determined.

Many synMuv B mutants show an enhanced RNAi (Eri) phenotype and PGL-1 misexpression in somatic cells, but some do not (Figure S1 and [11]–[13], [16], [17], [31], [33], [39], [40]). Even among the mutants that show enhanced RNAi and misexpress P granules, we noticed significant phenotypic differences, including strongly *vs.* weakly enhanced RNAi (Figure 1A), enhanced transgene silencing *vs.* transgene desilencing (Figure 1B, 1C), and large sparsely distributed *vs.* small densely clustered PGL-1 granules that are misexpressed in the intestine (Figure 1D). These differences suggest functional specializations among synMuv B proteins, and may reflect distinct activities from individual functional classes. To test this, we systematically surveyed and classified all synMuv B genes based on these phenotypes.

As shown in Figure S2, the phenotypic differences segregated coherently and could be used to classify synMuv B mutants into three distinct classes. Null or strong mutations in all but one of the seven DRM components strongly enhanced response to dsRNA causing a strong enhanced RNAi phenotype (close to 100% RNAi phenotype, Figure 1A), enhanced transgene silencing (Figure 1B) and caused the somatic misexpression of PGL-1 granules that were large and sparsely distributed around the nucleus of intestinal cells, as revealed by immunofluorescence imaging (Figure 1D). The only exception was *efl-1*/*E2F*, which was previously reported not to cause enhanced RNAi and PGL-1 misexpression [12], [16]. We also did not observe any phenotype upon inactivation of *efl-1*, suggesting that EFL-1 is redundant or may not participate in all the activities of the DRM complex.

In sharp contrast, null or strong mutations in the synMuv B heterochromatin class genes induced a noticeably weaker enhanced RNAi phenotype (60% or barely detectable RNAi phenotypes, Figure 1A), transgene *desilencing*, which is exactly the opposite from the transgene silencing phenotype of the DRM complex factors (Figure 1C), and somatic misexpression of PGL-1 granules that were much smaller and densely clustered around the nucleus in intestinal cells (Figure 1D). The only exception is *met-1*, which had no detectable phenotype, suggesting a specific involvement of H3K9 methylation (catalyzed by MET-2) but not H3K36 methylation (catalyzed by MET-1) [31], [41], [42]. One interesting feature of this class is the decoupling between feeding RNAi efficiency and transgene silencing ability. It suggests that either these proteins inhibit a unique aspect of feeding RNAi, or they have additional roles in transgene silencing (see below).

Results for the synMuv B NuRD components were distinct from the other gene classes. Only transgene silencing and PGL-1 misexpression in the soma were assayed due to sterility/lethality associated with these mutants. RNAi inactivation of *mep-1* caused enhanced transgene silencing, whereas inactivation of *hda-1* or *let-418* had no effect, suggesting that MEP-1 may function separately in transgene silencing. As reported [17], inactivation of *let-418* and *mep-1* caused somatic PGL-1 misexpression. However, the misexpressed PGL-1 granules were small and densely clustered around the nucleus, as in the case of the heterochromatin class of synMuv B mutants but distinct from the DRM class mutants. Intriguingly, neither *hda-1* mutant nor *hda-1* RNAi treated worms showed detectable misexpression of PGL-1 in the soma (Figure 1D), suggesting that either the histone deacetylase activity of NuRD is not required, or MEP-1 and LET-418 function outside the context of NuRD in preventing germline gene expression in the soma (see below).

Among the less studied synMuv B genes, we found that *lin-15b* mutant worms showed strongly enhanced RNAi (Figure 1A), enhanced transgene silencing (Figure 1B), and misexpressed large PGL-1 granules that are sparsely distributed around the nucleus (Figure 1D), resembling mutants of the DRM complex (Figure 1E). On the other hand, inactivating *lin-65* led to weakly enhanced RNAi (Figure 1A), transgene desilencing (Figure 1C), and misexpressed small PGL-1 granules that are densely clustered (Figure 1D), resembling the synMuv B heterochromatin class (Figure 1E). Inactivation of *tam-1* caused weakly enhanced RNAi, transgene silencing, but no PGL-1 misexpression, and thus may function independently in inhibiting only RNAi (Figure 1E). No phenotype was observed upon inactivating *lin-36*, *rpb-11*, *E01A2.4* or *gei-4*, suggesting that they may not be involved in inhibiting RNAi or repressing germline genes in soma but rather may be involved in other aspects of vulva precursor cell specification (Figure 1E).

### Individual synMuv B classes distinctly repress RNAi and P granule genes

To test whether each synMuv B gene class differentially represses the expression of germline genes in somatic cells in general, we sought to identify molecular targets of each class. Given the enhanced RNAi and *pgl-1* misexpression phenotypes, we first inspected the microarray studies of *lin-35* mutant animals for somatic misexpression of known P-granule and RNAi genes [43], [44]. We focused on the larval stage one (L1 stage) microarray comparisons between wild type and *lin-35* null mutant animals because at that stage the germline has two quiescent cells and the soma has 550 cells. As verified in our analyses below, any misexpression of germline genes in somatic tissues at this stage would be more readily observed than at later stages, where a genetic ablation of the germline would be necessary. Because of the distinct features of enhanced RNAi and somatic misexpression of P granules in the various classes of synMuv B mutants, we expected that particular suites of germline components might be misexpressed in each class of synMuv B mutants.

*pgl-1* was dramatically upregulated at the L1 stage in two microarray studies of *lin-35/Rb* mutant larvae. In addition, three other genes known to encode P granule components, *glh-1*, *pgl-3* and *spn-4*, were also upregulated (Figure S3B), suggesting that the somatic misexpression of P granule components stems from transcriptional misregulation of germline genes in the somatic cells of the *lin-35/Rb* mutant. In fact, as shown in Figure S3A, out of the 307 genes that are upregulated in both L1 stage arrays, 28% (86 genes) were identified as more than 2-fold germline-enriched by microarray [45],representing a 4.6-fold enrichment over the entire genome (1584 genes, 8.3%). Similarly, 18.6% (57 genes) of the *lin-35* L1 up genes were identified as germline-specific or germline-enriched by SAGE [46], a 3.3-fold enrichment over the entire genome (1063 genes, 5.6%). These analyses point to a striking preference of germline-enriched genes among the upregulated genes in these synMuv B mutants, which highlights the targeting of synMuv B genes to the repression of germline genes in somatic cells.

We next looked for RNAi factors that were upregulated, which may contribute to the enhanced RNAi phenotypes. None of the genes encoding canonical RNAi factors such as *rde-1* or *dcr-1* were upregulated in any of the arrays, consistent with previous studies [11]. However, we found several genes encoding less well studied RNAi factors to be upregulated (Figure S3B). Among them, *C16C10.3/wago-9*, *mut-2*, *drh-3*, *rde-4 and csr-1* are normally germline enriched [45], [46], and thus likely represent germline genes that are misexpressed in the soma. Four genes *C04F12.1*, *sago-2*, *rrf-2* and *drh-1* are not germline-enriched [45], [46], and thus represent ubiquitous RNAi factors that are upregulated.

We carried out real-time RT-PCR experiments to confirm the upregulation of these genes in *lin-35/Rb* mutants, and to test whether the same upregulations occur in mutants of other synMuv B classes. To specifically detect upregulation in the soma, the *glp-4(bn2)* temperature sensitive mutant was used, which ablates germline development at 25C. We were able to verify more than two-fold upregulation for 11 out of the 13 candidate somatically expressed germline genes in *glp-4 lin-35* mutants. Two genes, *csr-1* and *drh-1*, showed only marginal upregulation (less than two fold), and were not analyzed further.

**Figure 2:**
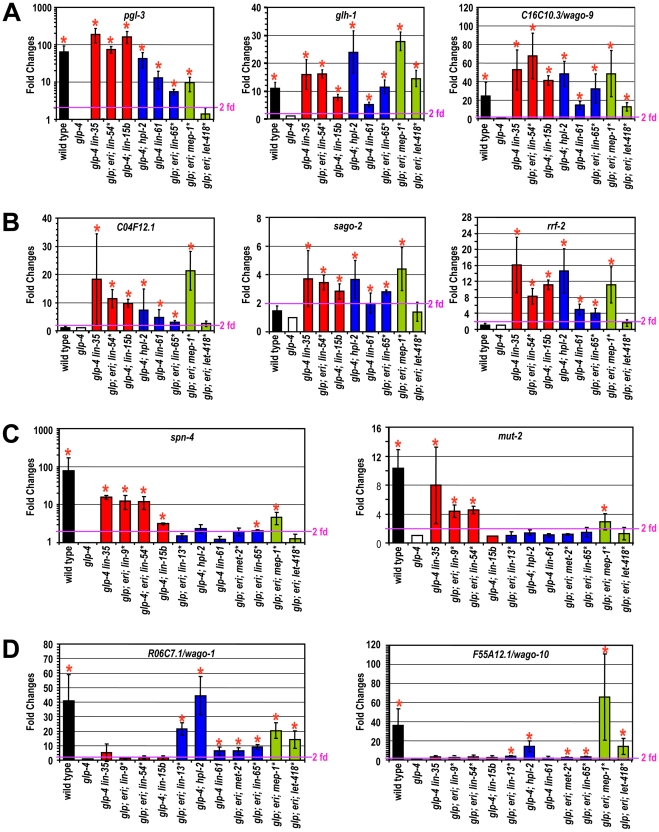
Different synMuv B classes distinctly repress P granule and RNAi genes in the soma. Real-time RT-PCR assays were performed using L4 stage worms with the indicated genotype or RNAi treatment. Expression levels were compared to that of *glp-4* worms and fold upregulations are plotted on the Y-axis. An asterisk indicates that the synMuv B gene was inactivated by RNAi in *glp-4(bn2); eri-1(mg366)* double mutant animals and expression levels were compared to that of vector control treated animals. Fold upregulations represent target expression in the soma, except for those in wild type animals, which represent the germline-enriched nature of the target gene. Asterisks represent greater than 2-fold mean fold change and with a *p*-value of less than 0.05 in two-tailed t-tests. Target genes were categorized into (A) germline-specific common targets, (B) ubiquitously expressed common targets, (C) DRM-specific targets, (D) synMuv B heterochromatin and MEP-1-LET-418 specific targets.

We tested whether the same upregulations occur when inactivating other synMuv B classes. Specifically, we individually inactivated known components of each class, either by genetic mutation or by RNAi knockdown, and measured the expression of these 11 genes in the soma. Of these eleven genes, seven were upregulated upon the inactivation of most components of all three synMuv B classes. These include the P granule genes *pgl-1*, *pgl-3* and *glh-1*, the germline enriched RNAi gene *C16C10.3/wago-9* (common germline targets), and all three ubiquitously expressed RNAi genes *C04F12.1*, *sago-2* and *rrf-2* (common ubiquitous targets) (Figure 2A, 2B and Figure S4). Somatic upregulations ranged from several fold to several hundred fold, but for a given gene, the extent of upregulation was largely similar among different synMuv B mutants. Interestingly, despite no enhanced RNAi or PGL-1 misexpression phenotypes, inactivation of *efl-1* induced upregulation of *glh-1*, *pgl-3* and *C16C10.3/wago-9* (Figure S4), suggesting that EFL-1 participates in the repression of some but not all DRM target genes.

Four germline-enriched genes, *spn-4*, *mut-2*, *drh-3* and *rde-4*, showed DRM-specific upregulation (DRM-specific targets) (Figure 2C and Figure S4). Compared to *glp-4* control, these genes were upregulated 4–16 fold in *lin-35 glp-4* mutants and upon RNAi knockdown of *lin-9*, *lin-54* or *lin-52* in *eri-1; glp-4* mutants. *spn-4* was also upregulated in *glp-4* worms treated with *efl-1(RNAi)*. Consistent with its classification as a DRM class mutant based on P granule morphology and enhanced RNAi, inactivation of *lin-15b* caused upregulation of all seven common targets, and a DRM-specific target *spn-4* (Figure 2C). None of these DRM-specific target genes were upregulated in *mep-1* or *let-418* inactivations (by RNAi in *eri-1; glp-4* mutants), nor in any of the synMuv B heterochromatin inactivations (in *glp-4; hpl-2* or *glp-4 lin-61* mutants, or upon RNAi knockdown of *lin-13* or *met-2* in *eri-1; glp-4* mutants) (Figure 2C and Figure S4). These results suggest that somatic repression of these genes solely depends on the DRM complex but not the synMuv B heterochromatin factors or MEP-1-LET-418 proteins.

The DRM-specific upregulations prompted us to look for genes that might be specifically upregulated in synMuv B heterochromatin or *mep-1-let-418* mutants. We focused on germline-enriched Argonaute genes that were not upregulated in *lin-35* microarrays because of the central position of Argonaute genes in small RNA pathways. A limited survey of these genes showed that *R06C7.1/wago-1* and *F55A12.1/wago-10* were significantly upregulated in almost all inactivations of the synMuv B heterochromatin class genes and *mep-1* & *let-418* (Figure 2D and Figure S4). In contrast, no significant upregulation of these Argonaute genes was detected in any of the DRM class synMuv B gene inactivations (Figure 2D and Figure S4). Therefore, the synMuv B heterochromatin class and MEP-1-LET-418 proteins are uniquely required to repress a specific set of germline-enriched RNAi factors in somatic cells.

We also extended the gene expression analyses to the remaining synMuv B genes. Inactivation of either *lin-15b* or *lin-65* led to the misexpression of common germline and common ubiquitous targets (Figure 2A, 2B). However, as shown in Figure 2C and 2D, inactivating *lin-15b* caused upregulation of *spn-4* (DRM -specific target), but not *R06C7.1/wago-1* or *F55A12.1/wago-10* (heterochromatin- and MEP-1&LET-418-specific targets). In contrast, inactivation of *lin-65* led to the upregulation of *R06C7.1/wago-1* and *F55A12.1/wago-10*, but only very marginal upregulation of *spn-4* and no upregulation of the other three DRM-specific targets. These gene expression patterns again place *lin-15b* into the DRM class genes while *lin-65* into the heterochromatin class, consistent with the previous phenotypic classification.

For synMuv B genes that did not show PGL-1 misexpression or enhanced RNAi phenotypes in the phenotypic classification, most of the inactivations led to no or very modest upregulations of these P granule or RNAi factors (Figure S4). The only exception is *gei-4*, whose inactivation led to the upregulation of several target genes. However, the most impressive upregulations observed were for the ubiquitous common targets, suggesting that *gei-4* may be more specifically involved in the repression of ubiquitous targets.

In summary, the three classes of synMuv B proteins function to repress overlapping sets of P granule and RNAi genes. The different spectra of misexpression suggest overlapping as well as non-overlapping functions among individual classes, and may underlie the distinct enhanced RNAi and transgene silencing phenotypes, and the distinct somatic P granule architectures observed in these mutants.

### The Mec complex, not NuRD, prevents germline gene expression in somatic cells

**Figure 3:**
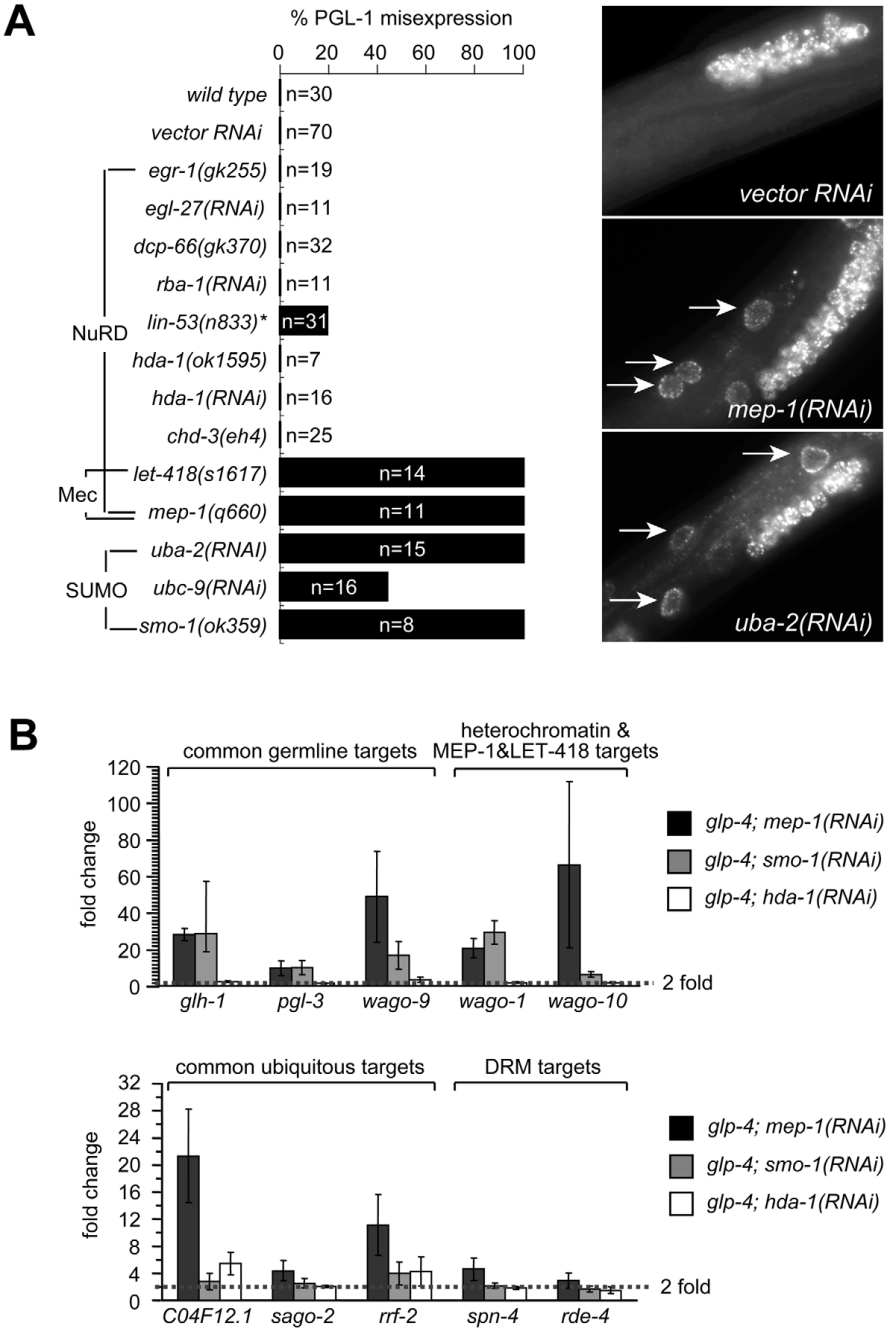
MEP-1 and LET-418 function as the Mec complex to prevent somatic germline gene expression by mediating the repressive effect of sumoylation. (A) SUMO, but not NuRD, is required to prevent somatic PGL-1 misexpression. Mixed stage animals with the indicated genotype or RNAi treatment were subjected to anti-PGL-1 immunostaining, and the percentages of animals showing somatic expression were plotted. n equals the total number of animals being assayed. Images of anti-PGL-1 stained animals were shown on the right. Arrows point to misexpressed PGL-1 granules in the intestine. Note the densely clustered small granules in RNAi treated animals. (B) SUMO, but not NuRD, is required to repress other germline-specific targets. Real-time RT-PCR assays were performed using L4 stage *glp-4* worms with the indicated RNAi treatment. Expression levels were compared to that of *glp-4* worms treated with control RNAi, and fold upregulations were plotted on the Y-axis. Fold upregulations represent target expression in the soma.

The observation that loss of *hda-1*, one of the catalytic subunits of the NuRD complex, does not induce somatic PGL-1 misexpression suggests that the entire complex is not required. To discern which NuRD subunits might be required, we surveyed the annotated *C. elegans* homologues of the NuRD complex for somatic P granule misexpression. We tested loss of function mutations and gene inactivations by RNAi of *egr-1* and *egl-27* (MTA homologues), *dcp-66* (p66 homologue), *chd-3* (*let-418* Mi2 paralogue) and *rba-1* (*lin-53* RBBP4 paralogue). We detected no somatic PGL-1 misexpression in any of the inactivations (Figure 3A), suggesting that NuRD is not involved. Consistent with this, loss of *lin-53*, a shared component between the DRM complex and the presumed NuRD complex, caused PGL-1 misexpression with a pattern that resembles that in the DRM mutants and differs from that in *mep-1* or *let-418* mutants. Thus, MEP-1 and LET-418 appear to function independently of NuRD in preventing somatic PGL-1 expression.

Recent work in flies has revealed that dMEP-1 and dLET-418 form a stable complex named dMec, which recognizes SUMO modifications on transcription factors and carries out transcriptional repression [47], [48]. *C. elegans* MEP-1 was reported to recognize and bind SUMO modified transcription factor LIN-1 and help repress LIN-1 target genes [49]. To test whether repression of PGL-1 misexpression required SUMO and a homologous Mec complex, we tested whether the SUMO pathway is required. Indeed, inactivation of the genes encoding the SUMO-activating enzyme (*uba-2*), the SUMO conjugation enzyme (*ubc-9*), and SUMO itself (*smo-1*) all caused somatic PGL-1 misexpression (Figure 3A). More importantly, the misexpressed PGL-1 form small granules that are densely clustered around the nucleus, identical to those in *mep-1* and *let-418* mutant worms (Figure 3A). This result strongly supports the idea that in worms, MEP-1 and LET-418 prevent somatic PGL-1 misexpression as subunits of the Mec complex that requires SUMO modification of target transcription factors or chromatin factors, rather than as components in a NuRD complex.

We next examined whether the SUMOylation machinery also regulates the other germline RNAi components that are responsive to *mep-1* and *let-418*. Using real-time RT-PCR, we tested the effect of *hda-1* and *smo-1* on the repression of other target genes. As shown in Figure 3B and Figure S4, loss of *smo-1*, but not *hda-1*, caused somatic misexpression of *glh-1*, *pgl-3* and *C16C10.3/wago-9* (common germline targets) as well as *R06C7.1/wago-1* and *F55A12.1/wago-10* (synMuv B heterochromatin and MEP-1 and LET-418-specific targets). The same treatment did not lead to the upregulation of *spn-4*, *rde-4*, *mut-2*, and *drh-3* (DRM-specific targets), indicating that *smo-1* is specifically required for the repression of MEP-1/LET-418 target genes. Interestingly, *smo-1(RNAi)* failed to induce the upregulation of *C04F12.1* and *sago-2*, and only weakly upregulated *rrf-2* (common ubiquitous targets), suggesting that the SUMO pathway may be specifically required to repress germline genes. Inactivation of *hda-1* by RNAi did not lead to significant upregulation of any of the germline genes tested (Figure 3B and Figure S4), again supporting that the NuRD complex is not involved in repressing germline targets in the soma. On the other hand, *hda-1(RNAi)* did lead to a modest upregulation of ubiquitous targets *C04F12.1* and *rrf-2* (Figure 3B and Figure S4), suggesting that the NuRD complex may contribute to the repression of ubiquitous target genes.

The above results strongly suggest that it was the Mec complex, not the NuRD, that is required for preventing misexpression of germline genes in somatic cells. As in flies, the worm Mec complex may mediate transcriptional repressive effects of SUMO modifications on relevant transcription factors or chromatin factors.

### A synMuv B heterochromatin complex with unique functions

Phenotypic and gene expression analyses suggest that the class of synMuv B heterochromatin proteins likely function together, and in the case of transgene expression, act separately from the DRM or the Mec complexes. Consistent with acting separately from the DRM and the Mec complexes, members of the synMuv B heterochromatin class have distinct cytological localizations. GFP fusions to LIN-13 and HPL-2 form subnuclear foci that may correspond to compact heterochromatin [32], [34]. In contrast, DRM complex components and GFP fusions to MEP-1 or LET-418 showed uniform nuclear localization [26], [50]–[53].

We tested whether the synMuv B heterochromatin proteins physically function together cytologically and biochemically. We constructed a GFP fusion to the full length LIN-61/L3MBT protein and observed its subcellular localization. LIN-61::GFP rescued the Muv, enhanced RNAi and PGL-1 misexpression phenotypes of *lin-61(tm2649)* mutant animals, and thus is a functional fusion protein (data not shown). GFP expression was detected by western analysis in all stages of worm development. Expression was the highest in embryos, where GFP was visible in most cells.

**Figure 4:**
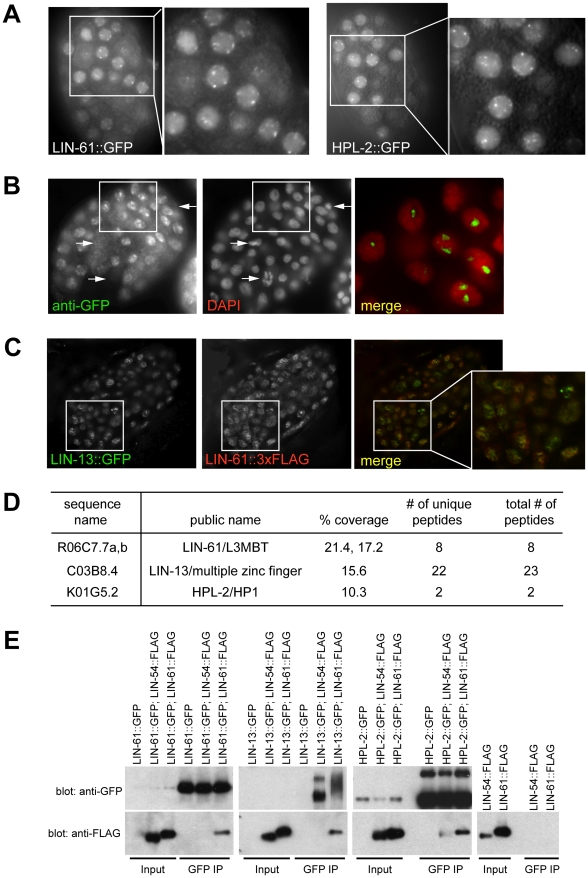
A synMuv B heterochromatin complex. (A) LIN-61::GFP fusion proteins concentrate at nuclear foci, similar to HPL-2::GFP foci, as shown by live fluorescent microscope images and zoomed in images of early embryos. (B) LIN-61::GFP foci fall into regions with low DAPI staining. Early embryos expressing LIN-61::GFP were costained with an anti-GFP antibody and DAPI. The merged image represents a zoom-in of the boxed region in the whole embryo and was contrast enhanced to highlight the localization of concentrated GFP foci in DAPI holes. Arrows point to LIN-61::GFP negative nuclei, which have condensed chromatin and are likely undergoing mitosis. (C) LIN-13::GFP and LIN-61::3xFLAG fusion proteins localize to overlapping nuclear foci. Embryos coexpressing both fusion proteins were costained with anti-GFP and anti-FLAG antibodies. A merged image that zooms into the boxed region in the whole embryo is also shown. (D) LIN-61::GFP interacts with LIN-13 and HPL-2. Anti-GFP immunoprecipitate from LIN-61::GFP expressing embryos was analyzed by mass spectrometry. Peptide coverage data for synMuv B heterochromatin class proteins were shown. (E) LIN-13::GFP and HPL-2::GFP interact with LIN-61::3xFLAG protein in co-IP western assays. Only HPL-2::GFP showed interaction with LIN-54::3xFLAG but the interaction was weaker compared to its interaction with LIN-61::3xFLAG. Each input lane represents 0.4% of material used in IP.

LIN-61::GFP showed nuclear localization with concentrated foci, just like LIN-13::GFP and HPL-2::GFP (Figure 4A). To look at the relationship between LIN-61::GFP foci and DNA, we costained LIN-61::GFP embryos with anti-GFP and DAPI. As shown in Figure 4B, anti-GFP antibody revealed foci resembling those formed by live GFP. Moreover, the most intensely stained GFP foci colocalize with areas of low DAPI staining, which is very similar to the reported pattern for HPL-2::GFP in interphase nuclei [32]. LIN-61::GFP was specifically depleted in nuclei with condensed chromosomes that are probably undergoing mitosis (Figure 4B), which was also observed for LIN-13::GFP (data not shown). The similar cytological localizations suggest that LIN-61 may colocalize with LIN-13 and HPL-2 in the nucleus. We thus generated a rescuing LIN-61::3xFLAG fusion gene (data not shown) and simultaneously visualized both LIN-61::3xFLAG and LIN-13::GFP fusion proteins in embryos by double immunostaining. As shown in Figure 4C, both LIN-61::3xFLAG and LIN-13::GFP localized to subnuclear regions that ranged from concentrated localization to discrete foci, and mostly coincided with poor DAPI staining (data not shown). Most importantly, in cells coexpressing both fusion proteins, FLAG and GFP signals largely overlapped, consistent with a colocalization. However, unlike HPL-2::GFP foci, which depend on LIN-13 and disappear in the absence of *lin-13*, LIN-61::GFP foci were not affected by the loss of *lin-13* (data not shown), suggesting a different mechanism of recruitment. Indeed, *C. elegans* LIN-61 was recently shown to directly bind to H3K9 methylation marks, just like HPL-2 [54], which may serve as the basis of colocalization.

To test whether LIN-61 physically interacts with other synMuv B heterochromatin proteins, we identified LIN-61-interacting proteins using large scale immunoprecipitation followed by mass spectrometry analysis. Embryos expressing LIN-61::GFP were used. Mass spectrometry analysis identified significant peptide coverage for LIN-13 and HPL-2, which was absent from a parallel IP using untagged control embryos (Figure S5). This indicates that LIN-13 and HPL-2 were present in the immunoprecipitants and thus likely physically interacted with LIN-61 (Figure 4D). Although peptides corresponding to additional proteins were identified (X. Wu and G. Ruvkun, unpublished), none corresponds to components of the DRM or Mec complex. These results suggest that synMuv B heterochromatin proteins LIN-61, LIN-13 and HPL-2 form a complex that is distinct from the DRM or the Mec complex.

The interactions between LIN-61 and LIN-13 as well as between LIN-61 and HPL-2 were confirmed using coimmunoprecipitation-Western analyses. To this end, in addition to the LIN-61::3xFLAG fusion described above, we also generated a rescuing LIN-54::3xFLAG fusion gene (data not shown). LIN-13::GFP or HPL-2::GFP fusion proteins immunoprecipitated the coexpressed LIN-61::3xFLAG protein (Figure 4E). LIN-61::GFP fusion protein also immunoprecipitated LIN-61::3xFLAG, suggesting that there are more than one LIN-61 protein present in the immunoprecipitant, presumably reflecting a higher order packaging potential of heterochromatin proteins either by the self-dimerization potential of LIN-61/L3MBT proteins or of other members of this complex [5], [55]. By contrast, the LIN-54::3xFLAG protein was not detected in the LIN-13::GFP or LIN-61::GFP precipitates, and only weakly detected in the HPL-2::GFP precipitate, again pointing to the existence of a distinct heterochromatin complex, which may only weakly interact with other synMuv B chromatin complexes such as the DRM complex.

It is intriguing that the synMuv B heterochromatin mutants desilence transgenes, despite having enhanced RNAi response. This contradicts the general positive correlation between RNAi efficiency and the ability to silence transgenes, where enhanced RNAi efficiency often leads to enhanced transgene silencing while a deficiency in RNAi response usually leads to desilencing of transgenes [14], [15], [56]. Our analysis of double mutants showed that the transgene desilencing phenotype of synMuv B heterochromatin mutants was epistatic to a range of other enhanced RNAi mutants that also silence transgenes. Inactivation of synMuv B heterochromatin class genes reversed the transgene silencing induced by *eri-1* mutations, DRM class synMuv B mutations and the natural silencing in the germline of wild type *C. elegans* worms (Figure S6 and data not shown). Thus, the heterochromatin class of synMuv B proteins is uniquely required for transgene silencing, likely downstream or in parallel to their effects on RNAi efficiency. Similar to repetitive transgenes, silencing of endogenous repetitive gene clusters in the genome may also require both the small RNA-mediated silencing and the downstream or parallel actions of these heterochromatin factors. It is possible that the presence of heterochromatin proteins at the repetitive loci may in turn recruit additional small RNA factors to continue small RNA-mediated silencing, as has been suggested in yeast for the spreading of heterochromatin [57], [58]. Thus, in addition to the transcriptional upregulation of RNAi factors, the loss of silencing at endogenous repetitive gene clusters and the release of small RNA factors such as Argonaute proteins to the exogenous RNAi pathway may also contribute to the enhanced RNAi effect in these heterochromatin mutants.

### DRM and Muv B heterochromatin complexes provide nonoverlapping functions

**Figure 5:**
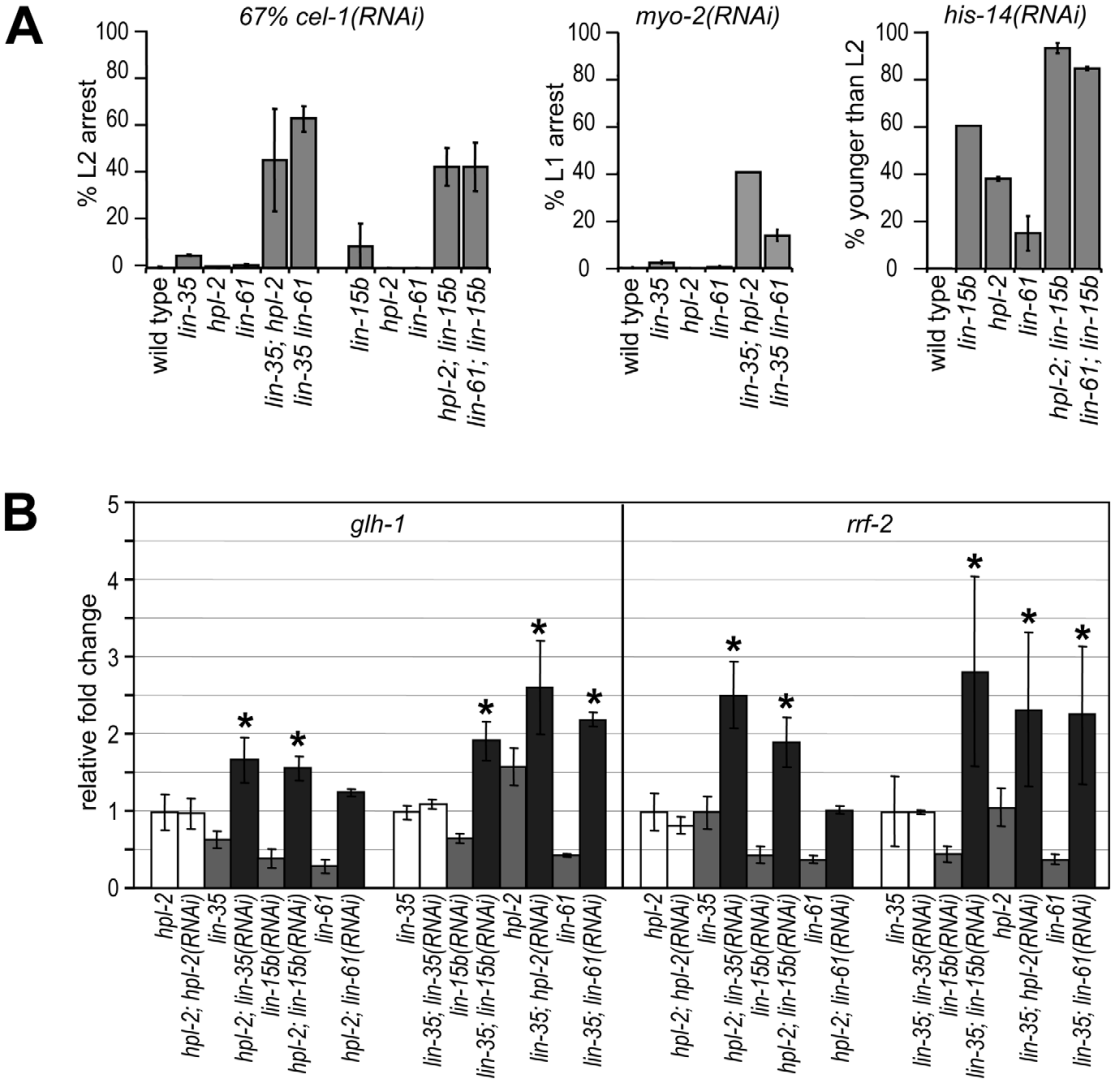
DRM and synMuv B heterochromatin class genes provide nonoverlapping functions. (A) DRM; synMuv B heterochromatin double mutant animals showed further enhanced RNAi efficiency than either single null mutant, as shown by the increased penetrance of RNAi phenotypes in double mutants (see Materials and Methods). (B) Additive upregulation of common targets in *glp-4; hpl-2* or *glp-4 lin-35* animals each treated with additional *synMuv B(RNAi)*. Real-time RT-PCR assays were performed on L4 stage animals and expression levels were each compared to those in respective vector control RNAi treated animals. Asterisks represent a *p*-value of less than 0.05 in two-tailed t-tests.

The phenotypic differences between DRM and heterochromatin classes of synMuv B mutants suggest that the two complexes may affect RNAi and the repression of germline genes in the soma through different pathways. We tested this possibility using a genetic approach, that is, genes that function in separate pathways should be additive to each other when testing null allele combinations. We first looked at feeding RNAi efficiency of *DRM; synMuv B heterochromatin* double mutants. To allow the detection of further enhanced RNAi, we used conditions where strong Eri mutants only showed a partial phenotype. We found that diluting 2 parts of the *cel-1(RNAi)* culture with 1 part of vector control RNAi culture dramatically reduced the RNAi response, reducing the nearly 100% L2 arrest phenotype in *lin-35* mutants to only ∼5%. Under this condition, *lin-35; hpl-2* and *lin-35 lin-61* double null mutants showed significantly stronger RNAi response (∼40% to ∼60% L2 arrest) compared to either single null mutant, indicating that the synMuv B heterochromatin mutations and *lin-35* are additive (Figure 5A, left panel). The same is true when using *myo-2(RNAi)* as tester: while 0% to 2% single mutants scored showed L1 arrest, over 14% to 25% of the double mutants showed L1 arrest (Figure 5A, middle panel). Consistent with *lin-15b* being classified with the DRM complex, additivity was also observed in *heterochromatin; lin-15b* double mutants using the diluted *cel-1(RNAi)* assay (Figure 5A, left panel) or *his-14(RNAi)* as tester (Figure 5A, right panel). Since the mutations assayed were all presumed null alleles, the additivity in double mutants suggests that the DRM and synMuv B heterochromatin genes function in separate pathways.

To see whether DRM and synMuv B heterochromatin proteins prevent gene misexpression via the same pathway or separate pathways, we assayed the misexpression of target genes that are commonly upregulated in both single mutants (Figure 5B). Compared to treatment with vector control RNAi, *glp-4; hpl-2* mutant animals treated with *lin-35(RNAi)* displayed higher levels of misexpression of *glh-1* (germline targets), as well as *rrf-2* (ubiquitous targets). More importantly, the observed higher levels of misexpression exceeded those in *glp-4 lin-35* mutant animals, thus represent true additivity. Similar treatment with *lin-15b(RNAi)* also led to significant higher levels of misexpression, whereas treatment with *hpl-2(RNAi)* or *lin-61(RNAi)* did not. Results for other common target genes were inconclusive; although higher mean levels of misexpression were detected upon *lin-35(RNAi)* or *lin-15b(RNAi)* treatment in *glp-4; hpl-2* mutant animals, the higher levels did not significantly exceed those in *glp-4 lin-35* or *glp-4; lin-15b* animals, respectively (data not shown), possibly due to the much milder upregulation from the *hpl-2* mutation than from the *lin-35* or *lin-15b* mutation and the incomplete knockdown of *lin-35* or *lin-15b* activity by RNAi. Conversely, *glp-4 lin-35* mutant animals treated with *hpl-2(RNAi)* or *lin-61(RNAi)* displayed higher levels of misexpression of *glh-1* and *rrf-2*, consistent with additivity. Interestingly, treatment with *lin-15b(RNAi)* also led to a significant higher misexpression in *glp-4 lin-35* mutants, suggesting that *lin-15b* likely has additional functions beyond the DRM complex. Overall, the observed additive upregulations suggest that even though the DRM complex and synMuv B heterochromatin complex repress a common set of genes, they do so by providing nonoverlapping functions.

### Inactivation of *mes-4* or *mrg-1* preferentially suppressed synMuv B heterochromatin mutants

Genetic suppressors of synMuv B mutations have emerged from screens for suppression of the Muv phenotype or screens for suppression of the enhanced RNAi phenotype [11],[13],[23]. Most of these suppressor mutations correspond to other chromatin factors that may counteract the action of synMuv B chromatin proteins. A few of the chromatin-related suppressors suppressed multiple phenotypes associated with synMuv B mutants [11], [13], [23], suggesting that they may function antagonistically at target loci with synMuv B proteins. Given that the DRM complex and the synMuv B heterochromatin complex likely provide non-overlapping functions, it is important to know whether these broad-spectrum suppressors counteract all synMuv B activities, including those provided by different complexes.

**Figure 6:**
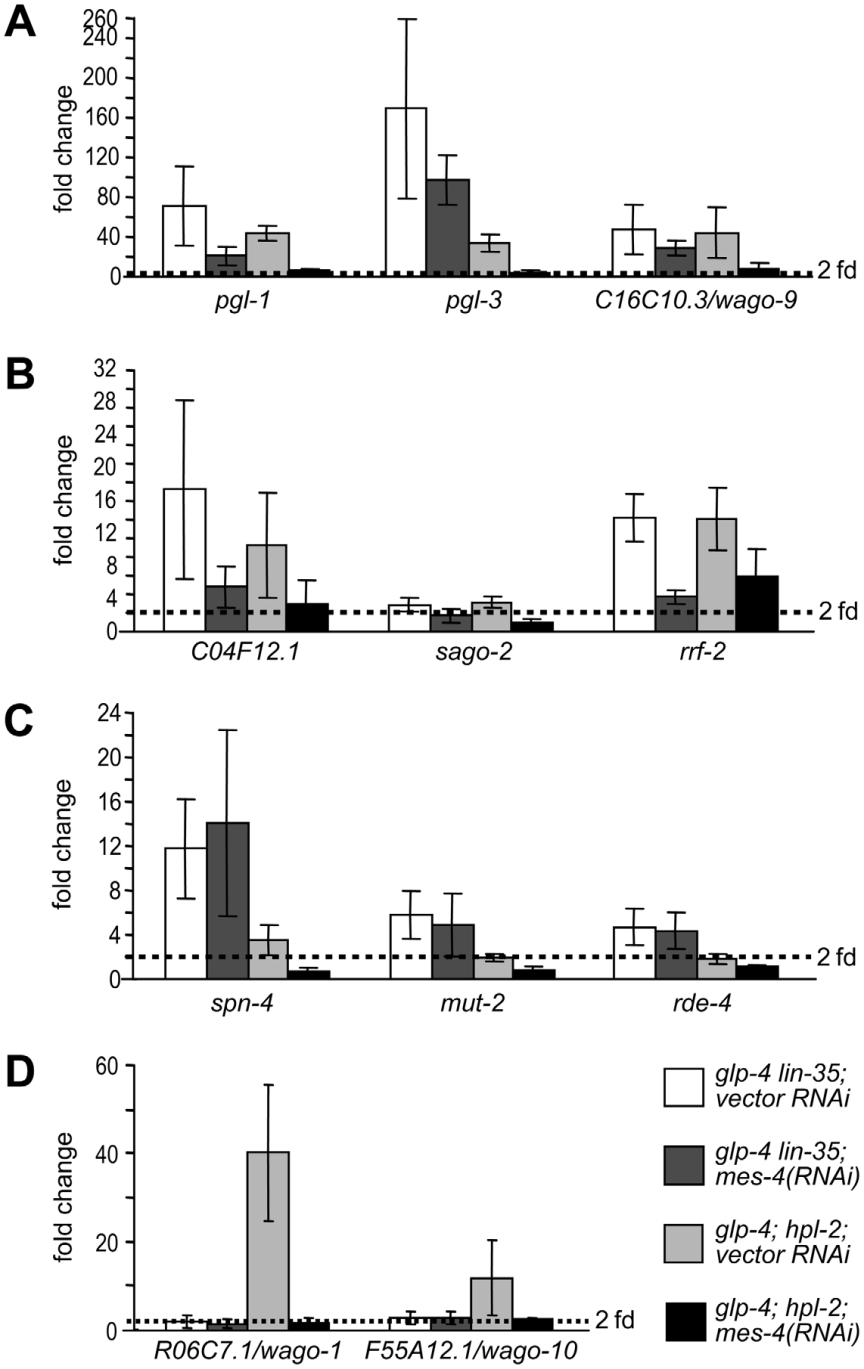
*mes-4/SET histone methyltransferase*, a synMuv B suppressor, preferentially counteracts the activity of the synMuv B heterochromatin class proteins. Real-time RT-PCR assays were performed using L4 stage worms with the indicated genotype and RNAi treatment. Expression levels of a given target gene were compared to that of *glp-4* worms treated with vector RNAi. Target genes were categorized into (A) germline-specific common targets, (B) ubiquitously expressed common targets, (C) DRM-specific targets, (D) synMuv B heterochromatin and MEP-1-LET-418 specific targets.

We addressed this question in the context of the somatically misexpressed P-granule and RNAi factors. We asked whether RNAi inactivation of two suppressors of both the PGL-1 misexpression and the enhanced RNAi phenotypes (*mes-4* and *mrg-1*) and two Eri-specific suppressors (*zfp-1* and *gfl-1*) can reverse the transcriptional upregulation observed in *lin-35* and *hpl-2* mutants. Inactivation of *zfp-1* or *gfl-1* did not suppress any of the transcriptional upregulation (data not shown), suggesting that they do not antagonize the function of synMuv B complexes at the level of target transcription. Instead, they might be directly involved in the RNAi process and thus function downstream of the upregulation of P granule and RNAi factor genes. In contrast, inactivation of *mes-4* (Figure 6A, 6B) and *mrg-1* (data not shown) suppressed the upregulation of the P granule and RNAi factor genes that are common to both DRM complex and synMuv B heterochromatin class. However, at least for the common germline targets (Figure 6A), the suppression was more complete in *hpl-2* mutants than in *lin-35* mutants, suggesting preferential suppression.

We explored this possibility by analyzing the effect of *mes-4* and *mrg-1* inactivations on target genes that are unique to individual synMuv B classes. As shown in Figure 6C, *mes-4(RNAi)* had no effect on the upregulation of DRM-specific targets *spn-4*, *mut-2* and *rde-4*. But rather, it fully suppressed the upregulation of synMuv B heterochromatin class-specific targets *R06C7.1/wago-1* and *F55A12.1/wago-10* (Figure 6D). *mrg-1(RNAi)* gave identical results (data not shown). The preferential suppression of target gene misexpression in the heterochromatin class synMuv B mutants further support functional specializations between individual synMuv B classes, and strongly suggest that the heterochromatin class synMuv B proteins specifically antagonize the activity of MES-4&MRG-1.

### Transcriptional misregulations contribute to the enhanced RNAi phenotype

**Figure 7:**
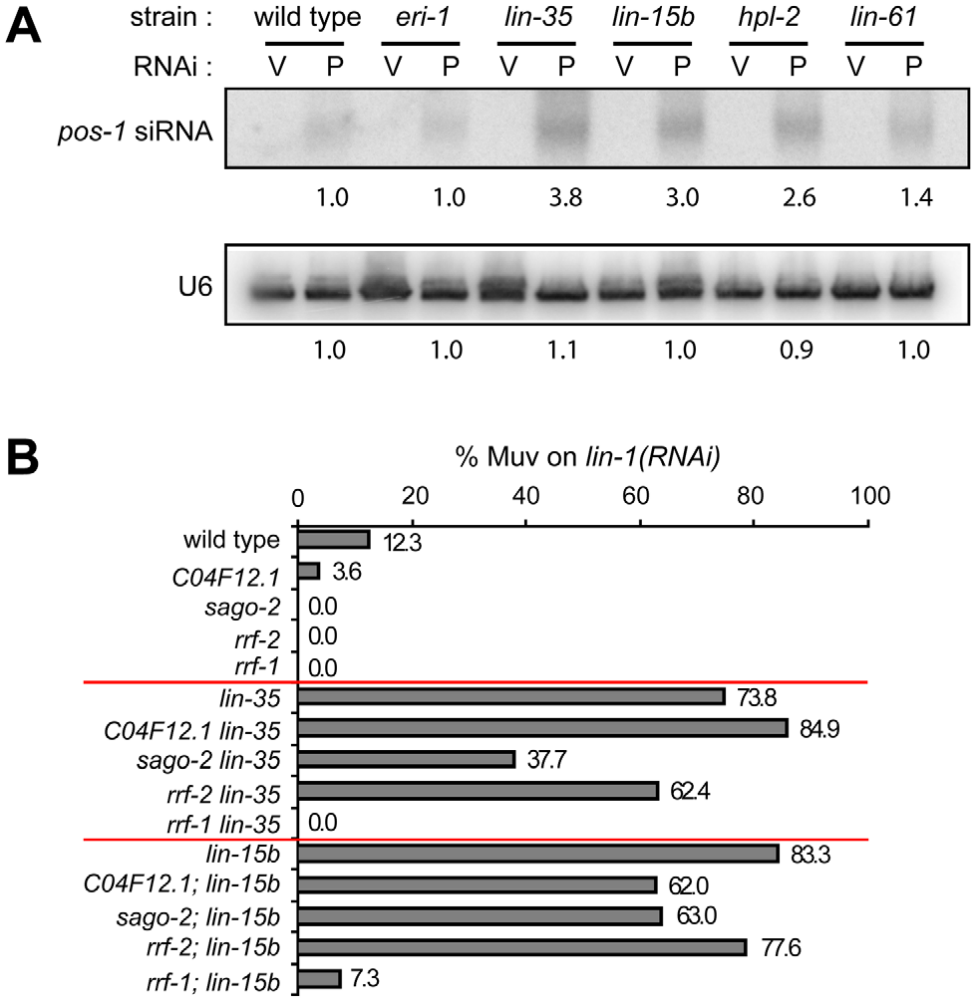
Transcriptional misregulations partially contribute to the enhanced RNAi response of synMuv B mutants. (A) Increased accumulation of siRNA levels in synMuv B mutants. Animals with the indicated genotype were fed vector RNAi (V) or *pos-1(RNAi)* food (P) and *pos-1* siRNA levels were measured by Northern blotting. U6 levels served as loading control. (B) Upregulated RNAi factors are partially required for enhanced RNAi. Indicated mutants were fed *lin-1(RNAi)* and the percentage of Muv animals were scored in the second generation.

Upregulation of RNAi factors was only observed in synMuv B mutants that exhibit enhanced RNAi, suggesting that the elevated levels of these RNAi factors contribute to the enhanced RNAi phenotype. We explored this possibility by two means. First, RNAi factors that are commonly upregulated in synMuv B enhanced RNAi mutants include three Argonaute proteins and an RNA-dependent RNA polymerase, all of which are predicted to promote siRNA accumulation. We measured the siRNA levels in synMuv B mutants after *pos-1(RNAi)*. *pos-1* siRNAs accumulated to significantly higher levels in synMuv B mutants than in wild type animals (Figure 7A). The increase correlates with the strength of enhanced RNAi response: 3.8- and 3.0-fold in the strong enhanced RNAi mutants *lin-35* and *lin-15b* vs. 2.6- and 1.4-fold in the weaker enhanced RNAi mutants *hpl-2* and *lin-61*. Increased siRNA levels are likely the result of transcriptional upregulation of RNAi factors, since it was not observed in *eri-1* mutants (Figure 7A).

Second, we asked whether the upregulated RNAi factors are required for the enhanced RNAi phenotype in synMuv B mutants. To this end, we introduced mutations of these RNAi factors into *lin-35* or *lin-15b* mutants, and asked if RNAi efficiency was attenuated in the double mutants. As shown in Figure 7B, mutations in *sago-2* and *rrf-2* mildly reduced RNAi efficiency in *lin-35* and *lin-15b* mutants. Mutation in *C04F12.1* also mildly reduced RNAi efficiency in *lin-15b* mutants. Since mutations of *sago-2* and *rrf-2* on their own had little or no effect on RNAi efficiency [59], [60], the observed effects are specific to synMuv B mutants. The strain carrying a mutation in *C16C10.3/wago-9* has weakly enhanced RNAi and was additive to the synMuv B mutations in the double mutant, and was thus not suitable for this analysis (data not shown). We also tested whether forced overexpression of these RNAi factors would be sufficient to cause enhanced RNAi. Overexpression of the RNAi factors individually using *sur-5* promoter-driven high copy transgenes did not lead to detectable enhanced RNAi (data not shown). When tried to overexpress all four factors, we did not achieve systemic overexpression, and the resulting strain was not Eri (data not shown). Overall, we conclude that transcriptional upregulation of RNAi factors likely contribute to the enhanced RNAi phenotype in synMuv B mutants, and that robustly enhanced RNAi response may require simultaneous upregulation of multiple RNAi factors.

## Discussion

We have shown that synMuv B class chromatin factors form three separate functional entities: the core DRM complex, the SUMO-recruited Mec complex and the synMuv B heterochromatin complex. Together, they inhibit the expression of germline and RNAi genes in the soma to in turn inhibit RNAi. Misexpression of germline genes in mutants lacking the heterochromatin class synMuv B proteins strictly requires the germline chromatin factors MES-4 and MRG-1, suggesting an intimate antagonistic interaction between them. In contrast, the misexpressions in DRM mutants only partially depend on MES-4 and MRG-1, and thus require additional master mediators for germline transcription. SynMuv B proteins inhibit RNAi by repressing the expression of RNAi factors as well as by other mechanisms. Our results point to a new means of regulating RNAi potency and illustrate multiple activities required for repressing germline gene expression in somatic cells (Figure S7).

### synMuv B proteins uniquely inhibit RNAi

The synMuv B class genes inhibit RNAi using distinct mechanisms from other *eri* genes. First, synMuv B mutations are phenotypically additive with *eri* mutations. *eri-1; DRM* and *eri-1; synMuv B heterochromatin* double mutants are even more enhanced in RNAi than either single mutant ([13] and XW, ZS and GR, unpublished). Second, synMuv B proteins affect distinct aspects of RNAi compared to proteins in the *eri-1* pathway. Unlike *eri-1* mutants, synMuv B mutants are not defective for the production of *eri-1*-dependent endo small RNAs (XW, ZS and GR, unpublished), and are normal for the *eri-1*-dependent nuclear localization of the NRDE-3/Argonaute protein (MC and HM, unpublished). synMuv B mutants, however, accumulate higher levels of siRNAs, which did not occur in *eri* mutants. Taken together, synMuv B proteins likely inhibit RNAi at a different step than Eri pathway proteins. Rather than losing endogenous small RNA processes and releasing shared factors to exogenous RNAi [61], [62], as proposed for the *eri* pathway, synMuv B proteins directly affect the expression levels of RNAi factors.

synMuv B proteins may repress the expression of yet to be identified RNAi factors in addition to those we have tested. This is particularly likely given that the upregulated RNAi factors studied here only partially mediate the enhanced RNAi phenotype. Indeed, several other *lin-35*-responsive genes were positives in previous screens for RNAi factors [56], [63]–[65]. Specifically, *cin-4*, *spd-5* and *lin-5* are required for dsRNA-mediated silencing of an RNAi sensor [56]. *rad-51* is required for RNAi-mediated transcriptional gene silencing of a transgene [63]. *rnp-8*, *kbp-3*, *rsa-1* and *vha-7* are required for tandem high copy transgene-induced cosuppression of homologous endogenous gene [64], while *ucr-2.3* is required for transposon silencing [65]. Intriguingly, six out of these nine genes encode proteins that are important for chromosome biology during cell division (*cin-4, spd-5, lin-5, rad-51, kbp-3, and rsa-1*), which again may reflect an intersection between chromatin regulators and RNAi machinery.

The role of synMuv B proteins in RNAi involves more pathways than the misexpression of RNAi factors, as the effects of chromatin go beyond transcriptional regulation. In support for this, many of the chromatin-related synMuv suppressors are required for efficient RNAi [11], suggesting that they may directly function in RNAi and their activities may be affected by synMuv B proteins. Among them, some may rescue the gene expression defects (*e.g.*, *mes-4* and *mrg-1*), making it complicated to discern their direct effects on RNAi. Others, however, may not rescue target gene misexpression in somatic cells (*e.g*., *zfp-1* and *gfl-1*), and thus are more likely to function directly in RNAi mediated silencing. Future work will be focused on establishing potential physical interactions between synMuv B proteins and their suppressors and possible functional regulations on one another.

Among synMuv B proteins, the DRM complex and the synMuv B heterochromatin complex each distinctively inhibit RNAi. This may involve the transcriptional regulation of common and unique RNAi factors (as shown in this paper), or functional regulation of specific chromatin-related RNAi factors. Further understanding requires the identification of RNAi-related transcriptional as well as functional targets that are specifically regulated by individual synMuv B complexes.

### Multiple pathways prevent the expression of germline-specific genes in somatic cells

Our gene expression analysis of retinoblastoma pathway mutants reveals transcriptional upregulation of a suite of germline-specific genes in somatic cells of these mutant animals. More importantly, we provided strong evidence pointing to nonredundant functions by multiple synMuv B complexes, including the distinct subcellular architectures of the misexpressed PGL-1 granules, the partially nonoverlapping spectra of misexpression, and the genetic additivity between them. Their differential interactions with MES-4 and MRG-1 support their assignment to distinct pathways that prevent somatic expression of germline genes (discussed later).

Inactivation of the insulin pathway or components of the CCT cytosolic chaperonin complex also cause misexpression of germline-specific genes in somatic cells [66]. Some of the dysregulation in the *daf-2* insulin pathway mutant may be through the inhibition of the MEP-1-LET-418 Mec complex as a result of PIE-1 misexpression [17], [66]. However, the somatic misexpressions in *daf-2* or *cct* mutants are much more modest than those in Mec mutants, and are hypothesized to involve other factors in addition to MES-4 and MRG-1 [66]. Thus, the insulin and cytosolic chaperonin pathways may represent a parallel mechanism for preventing germline gene expression in somatic cells. The relationship between *daf-2* and the DRM complex awaits more investigation.

The misexpression of germline-specific genes in somatic cells of animals lacking DRM function is extensive. Analogous microarray experiments in the soma of Mec, synMuv B heterochromatin, insulin and *cct* mutants will be necessary to disclose the full range of misexpression upon losing these other pathways. Ultimately, these misexpression profiles may help us understand the developmental and physiological phenotypes unique to each mutant.

### A refined model for repressing germline genes in the soma

During *C. elegans* embryogenesis, primordial germline cells (PGCs) and somatic cells are derived from the same mother cells and both inherit MES-4/histone methyltransferase, whose activity sets up a germline competent chromatin [41], [67]. MES-4 binds to genes that are expressed in the maternal germline, deposits RNA pol II-independent histone H3K36 trimethylation and marks them for germline expression in the current generation [41]. To prevent germline genes from expression in the soma, somatic cells require a mechanism to counteract the action of MES-4 until it gradually disappears at about the 100-cell stage.

We propose that the Mec and synMuv B heterochromatin complexes counteract MES-4 activity in somatic cells. This is supported by the fact that for those germline targets affected by these two complexes, MES-4 binds to *all* of them (Figure S3) [41], [67], [68] and mediates *all* their misexpressions. These two complexes may prevent the binding of MES-4 so that no new H3K36me3 marks can be deposited, or, they may prevent the recognition of MES-4-generated H3K36me3 marks. Our results that *mes-4* is also required for the misexpression of somatic targets, which are normally not bound by MES-4 [68], suggests that antagonizing MES-4 binding may be more likely. Thus, in the absence of Mec and synMuv B heterochromatin complexes, MES-4 may spread to these somatic genes and erroneously mark them as germline expressed. Directly analyzing the effects of Mec and synMuv B heterochromatin complexes on MES-4 localization at gene resolution by ChIP-chip and ChIP-seq techniques during embryogenesis will distinguish between these two possibilities.

It was hypothesized that MEP-1-LET-418 antagonize MES-4 activity via the NuRD complex, since PIE-1, which interacts with MEP-1-LET-418, inhibits the histone deacetylase activity of HDA-1 in NuRD [17]. However, our results strongly suggest that NuRD is not involved. Rather, MEP-1-LET-418 act as the Mec complex in response to SUMO modification. Potentially, this also involves the synMuv B heterochromatin complex, since LIN-61/L3MBT was also shown to mediate SUMO-triggered transcriptional repression [48].

It is unclear how the Mec and synMuv B heterochromatin complexes might prevent MES-4 binding. One possibility is that they change the structure and accessibility of chromatin, given that LET-418/Mi-2 is an ATP-dependent nucleosome remodeling factor while LIN-61/L3MBT and HPL-2/HP1 may cause higher order chromatin compaction [5], [55]. Alternatively, they may prevent MES-4 binding by recruiting other factors that erase the histone modifications recognized by MES-4 [69], [70]. Analyzing the states of histone modifications during normal repression *vs.* misexpression might help to understand the mechanisms.

The result that DRM-specific targets are not suppressed by *mes-4* inactivation indicates that the DRM complex also functions downstream of MES-4 or in a parallel pathway. The DRM complex might be recruited to these target loci by sequence specific DNA binding (*e.g.*, by EFL-1/E2F or by LIN-54/Mip120) and carry out repression. Indeed, among the 307 *lin-35* L1 stage up-regulated genes, 163 either contain E2F binding sites in their promoter [44], or were reported to be bound by LIN-54 in a ChIP-chip study [71], or both. Therefore, loss of DRM-mediated repression at target promoters may account for much of the misexpression that was observed. On the other hand, given the vast extent of gene misexpression in *lin-35* mutants, misexpression may also involve antagonizing master regulators of germline expression (*e.g.*, other germline chromatin factors). Identifying factors that specifically mediate the misexpressions in DRM mutants may uncover novel master regulators of germline expression.

It is interesting that the misexpressed germline genes are the most detectable in intestine and hypodermis, suggesting that these two tissues are the most prone to misexpression. This might be caused by a difference in the initial MES-4-MRG-1 activity levels or by a difference in the overall transcriptional activity and/or chromatin configuration among different tissues. These two are not mutually exclusive. Intestine and hypodermis are two tissues that remain replication competent even towards the end of developmental maturity (L4/adult molt) or even after that [72], [73]. As such, they may maintain a different chromatin state than other tissues, making them more susceptible to the action of germline chromatin regulators, and rely more on Mec and synMuv B heterochromatin complexes to prevent misexpression of germline genes.

### Relevance in general developmental and cancer biology

The function of LIN-35/Rb and other synMuv B chromatin factors to prevent germline gene expression in the soma is likely conserved in higher organisms. In *Drosophila*, misexpression of germ granule and small RNA factors were also detected in Rb mutant cells by microarray analyses [74] and in the brain tumors developed in l(3)mbt (*lin-61* homologue) mutant flies [75]. Future work to understand the antagonistic relationship between synMuv B complexes and MES-4-MRG-1 is crucial to understand the process of repressing germline genes in the soma during development. In addition, since the DRM complex functions beyond antagonizing MES-4-MRG-1, identifying the unknown components specific to the DRM pathway will likely uncover novel players that function in this process.

Besides regulating the cell cycle, mammalian Rb also promotes differentiation, and cancer cells are sometimes dedifferentiated [76]. The expression of germline-specific genes in somatic cells in worms and flies may be a form of dedifferentiation, and the role of synMuv B genes to prevent that may be important for tumor suppression. Consistent with this idea, the misexpressed germ granule and small RNA factors were shown to be required for the growth of the l(3)mbt mutant tumors [75]. Thus, the knowledge of how Rb and other synMuv B chromatin complexes function to prevent misexpression may contribute to our understanding of cancer formation and the identification of new pathway components may lead to the discovery of novel tumor suppressive and oncogenic pathways.

Finally, the explicit mapping of *C. elegans* LIN-35/Rb and other synMuv B chromatin factors to a regulatory pathway for repression of germline encoded small RNA pathways suggests that in some mammalian tumors, most especially the many that bear Rb mutations, there may be specific enhancement of small RNA pathways to empower the dedifferentiation of those tumors towards more multipotent germline-like cells. A specific prediction from our work is that one of the major transcriptional signatures of Rb tumors should be misexpression of germline small RNA cofactors and small RNAs themselves.

## Materials and Methods

### Worm strains and genetics

synMuv B alleles used in this study are listed in Figure S2. Other alleles used are *glp-4(bn2) I, smo-1(ok359) I, dcp-66(gk370) I, rrf-1(pk1417) I, rrf-2(ok210) I, C04F12.1(tm1637) I, sago-2(tm894) I, hT2[bli-4(e937) let-?(q782) qIs48] (I;III), szT1[lon-2(e678)] I;X, szT1 (I;X), dpy-18(e364) III, wago-9/C16C10.3(tm1200) III, dpy-17(e164) III, eT1(III;V), eri-1(mg366) IV, nT1[qIs51] (IV;V), unc-46(e177) dpy-11(e224) V, egr-1(gk255) V, chd-3(eh4) X*.

Transgenic strains *Is(sur-5::gfp) I*, JR672 [*wIs54(scm::gfp) V*], GR1403 [*sur-5::gfp I; eri-1(mg366) IV*], PD7271{*pha-1(e2123) III; ccEx7271[let-858::gfp pha-1(+)]*}, GR1402 [*eri-1(mg366) IV; wIs54 V*] and FR463 [*Is(HPL-2::GFP*+pRF4)] were previously described [32], [56].

The LIN-61::GFP translational fusion consists, in order, of a 2.9 kb *lin-61* genomic fragment containing the upstream intergenic sequence and coding region minus the STOP codon, an engineered sequence encoding a short peptide linker (GGAGGSAAA) followed by GFP sequence amplified from pPD99.77, and then a 244 bp *lin-61* 3′ genomic fragment starting immediately after the STOP codon. Extra chromosomal complex arrays were generated by injecting wild type animals with a mixture of 10 ng/ul LIN-61::GFP fusion plasmid+1 ng/ul P*myo-2*::NLS::mCherry (gift from J. Bai and J. Kaplan)+89 ng/ul sheared salmon sperm DNA. The LIN-61::3xFLAG fusion was generated by replacing the GFP sequence in LIN-61::GFP fusion construct with 3xFLAG sequence. LIN-54::3xFLAG fusion consists of a 2.6 kb *lin-54* genomic fragment containing the upstream intergenic sequence and coding region minus the STOP codon, the same short peptide linker followed by 3xFLAG sequence, and then a 413 bp *lin-54* 3′ genomic fragment starting immediately after the STOP codon. Extra chromosomal complex arrays were generated by injecting wild type animals with a mixture of 10 ng/ul 3xFLAG fusion plasmid+1 ng/ul P*mec-7*::mRFP+89 ng/ul sheared salmon sperm DNA. All integrated transgenic lines were obtained by UV irradiation, followed by six times backcrossing.

LIN-13::GFP was reported to be a functional fusion gene [34]. All other translational fusion genes were tested to be rescuing functional fusions (data not shown).

### RNAi clones

RNAi clones targeting *let-418*, *lin-13* and *lin-15b* were made by individually cloning genomic fragments corresponding to part of *let-418* exon 5 (1025 bp), part of *lin-13* exon 12 (727 bp) and part of *lin-15b* exon 5 (1053 bp) into the NcoI site of the Ahringer L4440 feeding vector. The other RNAi clones used were from the Ahringer or Vidal libraries.

### Transgene silencing assays

To measure *Is(sur-5::gfp)* expression, synchronized L1 animals of the appropriate genotype were treated with RNAi at 15C till the L3 stage of the same generation (P0 clones) or that of the second generation (F1 clones) before scoring. The same was done to measure *wIs54(scm::gfp)* expression, except that animals were cultured at 22C and GFP was scored at the L4 stage. Germline desilencing using PD7271 was assayed according to Kim *et. al* [56].

### Immunostaining and microscopy

For PGL-1 misexpression, mutant or RNAi treated animals were cultured at 22C for two generations (one generation for P0 RNAi treatment). PGL-1 immunostaining was performed as described [13] with some modifications. Freeze-cracked slides were immediately fixed at −20C in 100% methanol for 120 minutes, and then 100% acetone for 15 minutes. Larvae were stained with monoclonal anti-PGL-1 antibody (K76) at 1∶40 in PBSTB (PBS containing 0.1% Tween-20 and 1% BSA) overnight at 4C, followed by Alexa Fluor-conjugated goat anti-mouse IgM (Invitrogen) at 1∶100 in PBST for 1 hour at 25C. After washing with PBST, slides were mounted with Vectashield mounting medium with DAPI (H-1200) and analyzed using a Zeiss Axioplan2 microscope and Openlab imaging software. Immunostainings of LIN-61::GFP, LIN-13::GFP and LIN-61::3xFLAG in embryos were performed similarly. Rabbit polyclonal anti GFP (Invitrogen A11122) and Sigma M2 anti-FLAG were used at 1∶1000 and 1∶50, respectively. Alexa Fluor488-conjugated goat anti-rabbit Ig G and Alexa Fluor594-conjugated goat anti-mouse Ig G (Invitrogen) were both used at 1∶100.

To show detailed PGL-1 granule morphology (Figure 1D), stained animals were photographed using a Zeiss Imager Z1 microscope and AxioVision software. Images were each deconvoluted from a stack of fifteen optical sections using algorism provided by the software.

### Quantification of misexpression by real-time PCR

Synchronized L1 stage wild type, *glp-4*, and *synMuv B; glp-4* animals were cultured at 25C till L4 stage before collection. For one generation RNAi due to sterility (*let-418 RNAi, smo-1 RNAi, hda-1 RNAi*), L1 stage *glp-4* animals were dropped on RNAi food and allowed to grow at 25C till L4 stage. For all other experiments involving RNAi treatment, synchronized L1 stage *glp-4* animals were dropped onto RNAi food and cultured at 15C till gravid adults with plateful of eggs. Adults and F1 larvae were washed off, leaving the eggs behind. These eggs were allowed to hatch at 25C for 3 hours. The newly hatched larvae were washed off, transferred onto new plates with the same RNAi food and allowed to grow to the L4 stage before collection.

Total RNA was isolated using Trizol (Invitrogen) and cDNA was synthesized using Superscript III reverse transcriptase (Invitrogen). Negative control without reverse transcriptase was performed on a pooled sample of all the RNA samples under study. Real time PCR reactions were performed using SYBR green (BioRad). At least three biological replicate samples were tested in triplicate. For each primer pair, a standard curve was generated using serial dilutions of a pooled sample of all cDNA templates involved. Relative quantities were deduced using the standard curve. Fold changes of relative quantities were calculated and normalized to *rpl-32* and *act-1*. Fold differences for *rpl-32* and *act-1* were less than two fold. Primer sequences are available upon request.

### Immunoprecipitation

Embryos were collected by hypochlorite treatment of gravid adults and dropped into liquid nitrogen to freeze. Frozen pellets were freeze ground to fine powder using a mortar and pestle and 1∶6 resuspended in 50 mM HEPES pH 7.6, 140 mM KCl, 10 mM NaCl, 1 mM MgCl_2_, 1 mM EDTA, 10% glycerol, 0.1% β-octylglucoside, 0.5 mM BME, Complete Protease tablets (Roche), 2 mM PMSF. Lysates were cleared by two centrifugations each at 15000× *g* for 20 min. Immunoprecipitation was performed using monoclonal anti-GFP clone 3E6 (Invitrogen) coupled to Affy ProA agarose beads (BioRad) at 4C for 2 hours. Immunoprecipitates were washed using the same buffer. Bound proteins were eluted using 0.1 M glycine (pH 2.5), and precipitated using TCA. Pellets were washed with acetone, resuspended in 50 mM NH_4_HCO_3_ and sent for mass spectrometry.

Immunoprecipitations for co-IP-Western analyses were performed similarly, except that 50 ul packed embryos were used and bound proteins were eluted from the anti-GFP matrix by boiling in PAGE gel sample loading buffer. Eluted proteins were analyzed by SDS-PAGE and Western analyses. Roche anti-GFP (cat#11814460001), Sigma M2 anti-FLAG were used as primary antibodies, Pierce peroxidase-conjugated goat anti-mouse IgG antibody (cat#31430) and Pierce SuperSignal West Pico Extended Duration chemiluminescent detection kit (cat#34076) were used.

### RNAi feeding assay for additive Eri

For the diluted *cel-1(RNAi)* assay, OD_600_ matched *cel-1(RNAi)* and vector RNAi cultures were mixed at 2∶1 ratio by volume before seeding plates. First day gravid adults reared on OP50 food at 20C were exposed to RNAi food for 24 hours, then transferred to new plates with the same food for a 4-hour egg lay. Afterwards, adults were taken off the plate and laid embryos were allowed to hatch and grow at 20C. L2 arrest was scored when animals of the same genotype treated with only vector RNAi reached adulthood.

For the *myo-2(RNAi)* and *his-14(RNAi)* assays, synchronized L1 animals were treated with RNAi at 20C and were scored when animals of the same genotype reached adulthood on vector RNAi.

### Northern blotting

Synchronized L1 animals were dropped onto *pos-1(RNAi)* or vector RNAi food and cultured at 20C until gravid adults with plateful of eggs. Adults were collected and total RNA was isolated using Trizol (Invitrogen). 60 ug of total RNA were used for each sample. *pos-1* siRNA was detected using body-labeled *pos-1* probe (Ambion MaxiScript kit). The stripped blot was reprobed with an end-labeled U6 oligo probe. Hybridization signals were analyzed using ImageQuant software and normalized to wild type samples.

## Supporting Information

Figure S1 Summary of synMuv B gene classes based on molecular and biochemical associations and previously reported enhanced RNAi and PGL-1 misexpression phenotypes.
(TIF)

Figure S2 Summary of enhanced RNAi and PGL-1 misexpression phenotypes for synMuv B gene inactivations. Enhanced RNAi: enhanced response to *hmr-1(RNAi)*, *unc-73(RNAi)* and *dpy-13(RNAi)*. Transgene silencing: enhanced silencing of transgene arrays (*sur-5::gfp*, *scm::gfp* and *mgIS30*). Transgene desilencing: desilencing of transgenes arrays (*sur-5::gfp* and *scm::gfp*) in *eri-1(mg366)* background. PGL-1 misexpression: somatic misexpression of PGL-1 detected by anti-PGL-1 antibody staining.
(TIF)

Figure S3 (A) Extensive somatic expression of germline-specific genes in *lin-35* mutant animals as revealed by published microarray experiments. (B) Summary of P granule and RNAi genes that were upregulated in published microarray experiments.
(TIF)

Figure S4 Summary of target upregulations resulted from synMuv B gene inactivations as measured by real-time RT-PCR assays.
(TIF)

Figure S5 LIN-61::GFP associated proteins as revealed by silver-staining with comparison to anti-GFP western.
(TIF)

Figure S6 synMuv B heterochromatin class proteins are required for transgene silencing. (A) Fluorescent microscope images showing the desilencing effect of *lin-61(RNAi)* on different scenarios of transgene silencing (*eri-1*-induced, DRM-induced, and natural germline silencing). (B) Summary of transgene desilencing phenotypes upon RNAi inactivation of synMuv B heterochromatin class genes as measured by GFP fluorescence for transgene expression. −: no desilencing effect detected compared to vector RNAi control. +/− to +++: different levels of desilencing, ranging from marginal to strong.
(TIF)

Figure S7 A model for repression of germline P granule and RNAi components in somatic cells by synMuv B genes.
(TIF)
