# Supplementary figures and images for: Repression of Germline RNAi Pathways in Somatic Cells by Retinoblastoma Pathway Chromatin Complexes
Source: PLoS Genet. 2012 Mar 8;8(3):e1002542. doi: 10.1371/journal.pgen.1002542 (PMC3297578; doi:10.1371/journal.pgen.1002542)

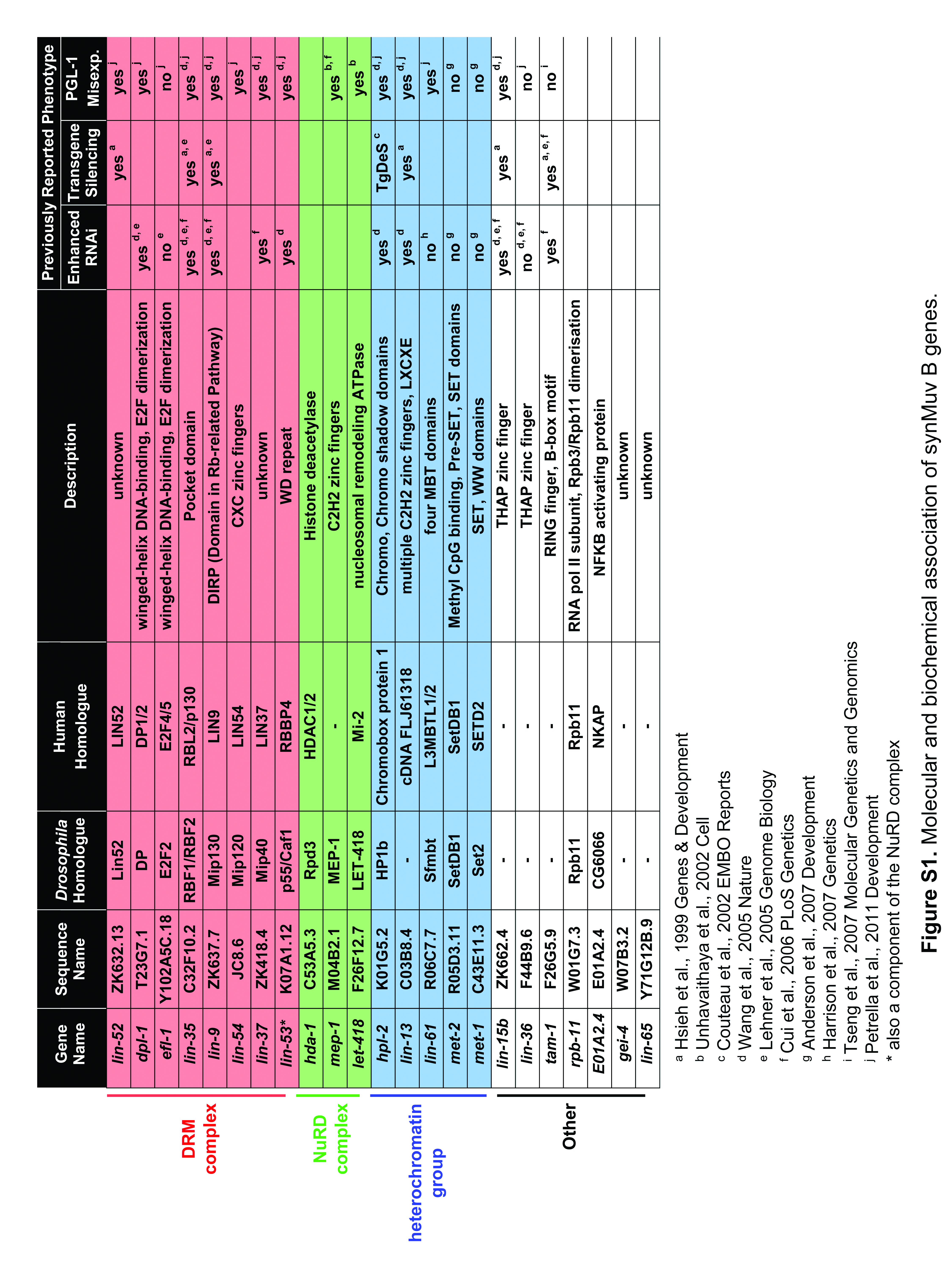

Supplement: Figure S1 — Summary of synMuv B gene classes based on molecular and biochemical associations and previously reported enhanced RNAi and PGL-1 misexpression phenotypes. (TIF) [file pgen.1002542.s001.tif]

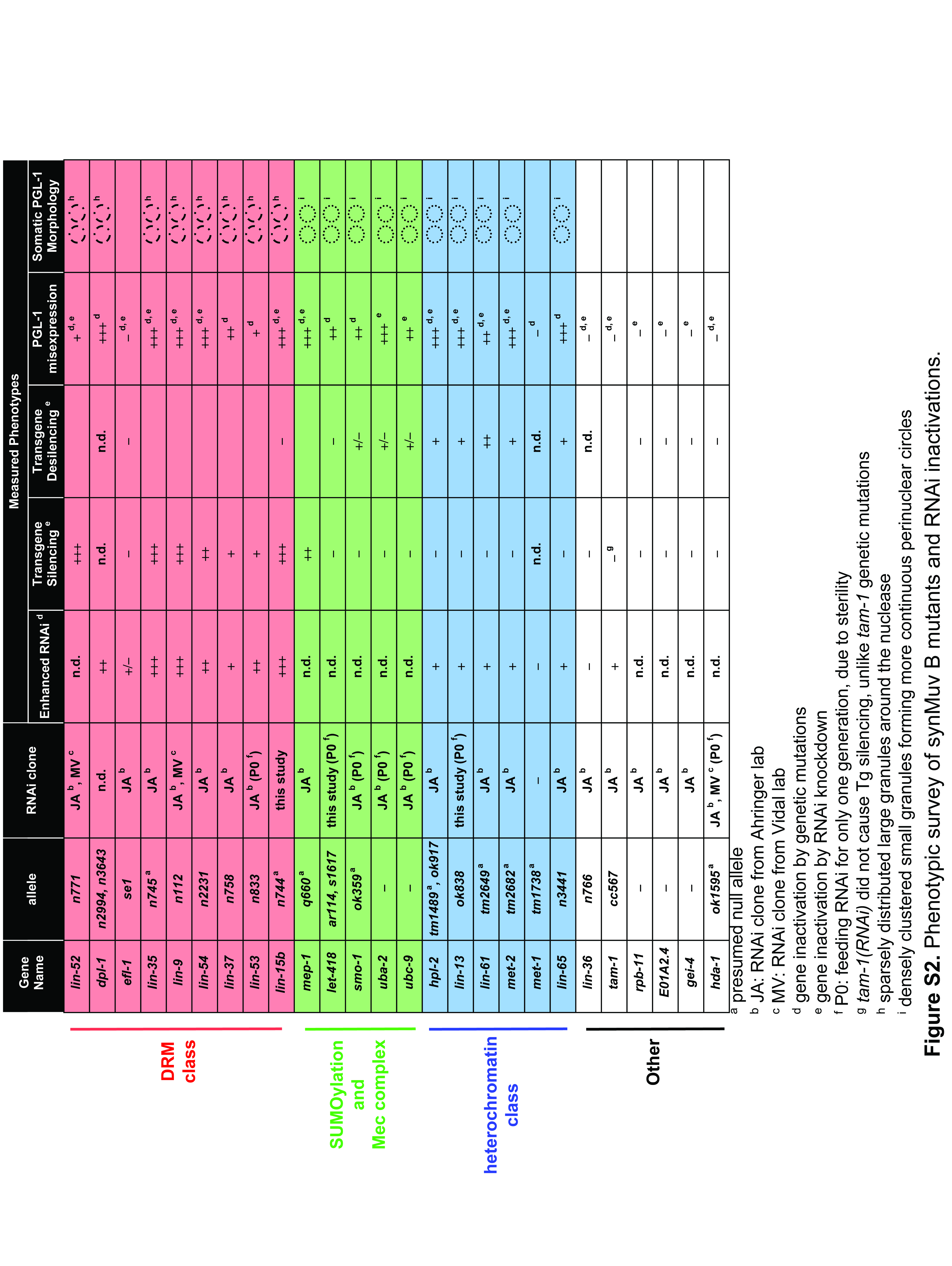

Supplement: Figure S2 — Summary of enhanced RNAi and PGL-1 misexpression phenotypes for synMuv B gene inactivations. Enhanced RNAi: enhanced response to hmr-1(RNAi), unc-73(RNAi) and dpy-13(RNAi). Transgene silencing: enhanced silencing of transgene arrays (sur-5::gfp, scm::gfp and mgIS30). Transgene desilencing: desilencing of transgenes arrays (sur-5::gfp and scm::gfp) in eri-1(mg366) background. PGL-1 misexpression: somatic misexpression of PGL-1 detected by anti-PGL-1 antibody staining. (TIF) [file pgen.1002542.s002.tif]

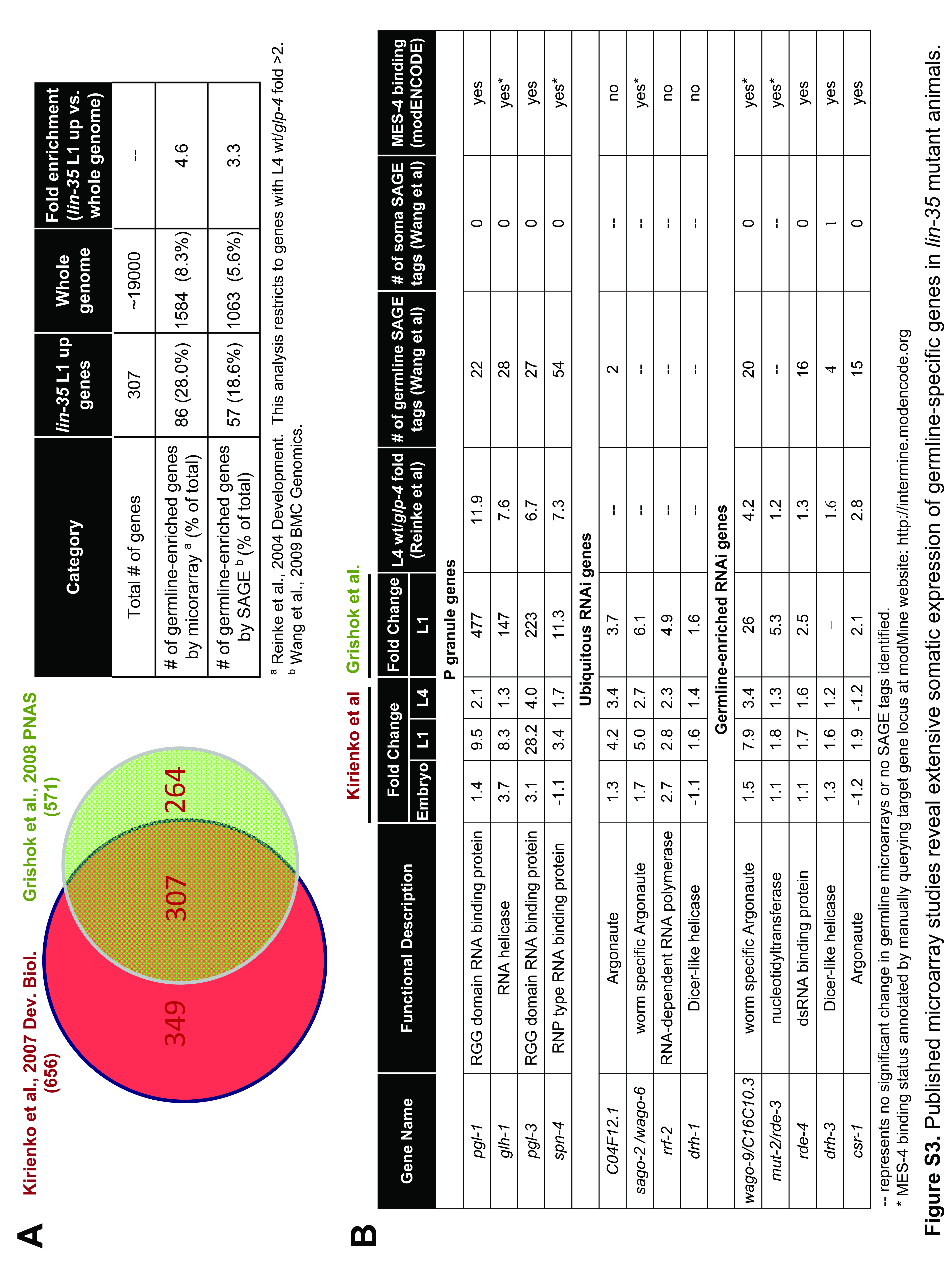

Supplement: Figure S3 — (A) Extensive somatic expression of germline-specific genes in lin-35 mutant animals as revealed by published microarray experiments. (B) Summary of P granule and RNAi genes that were upregulated in published microarray experiments. (TIF) [file pgen.1002542.s003.tif]

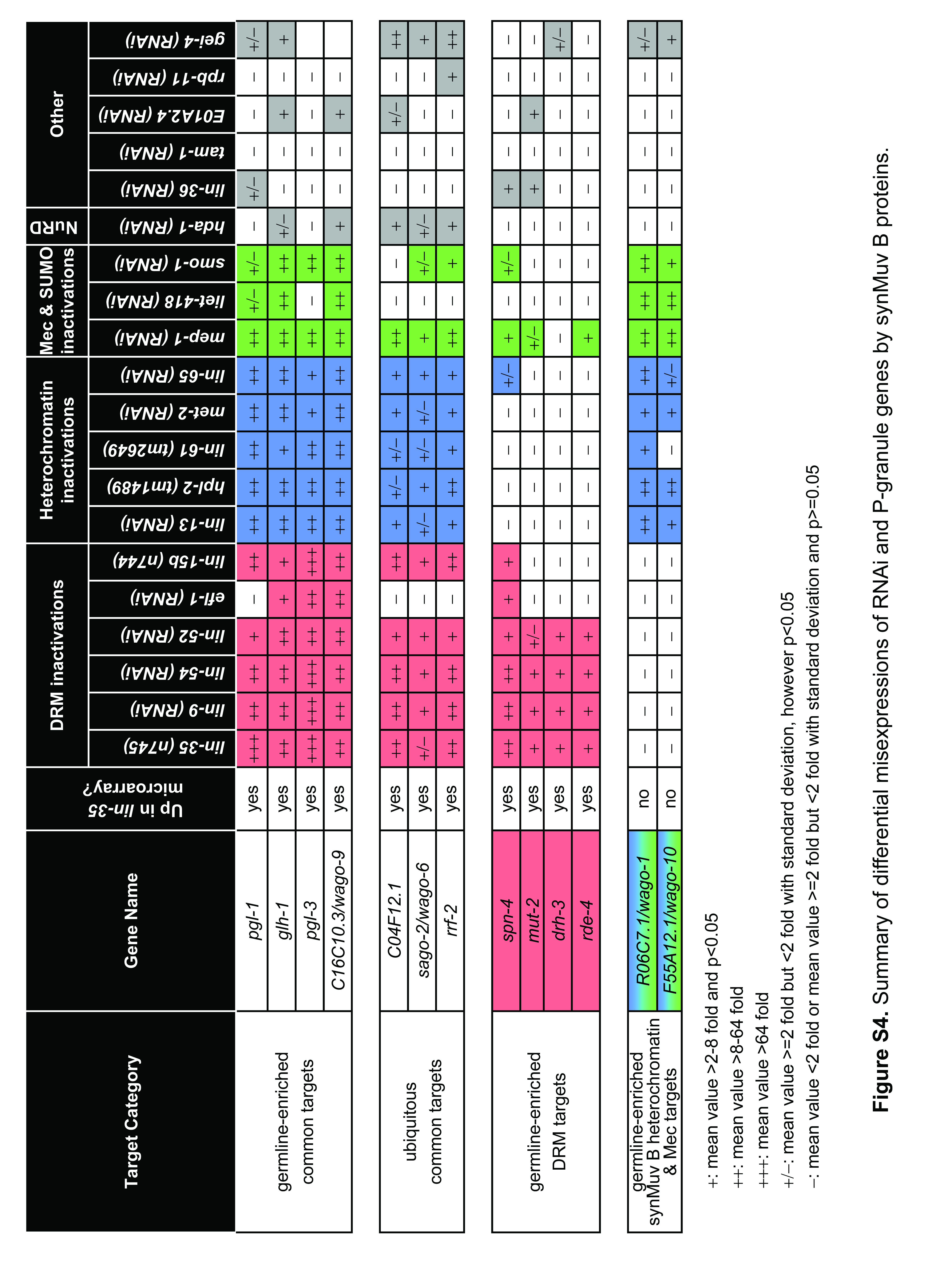

Supplement: Figure S4 — Summary of target upregulations resulted from synMuv B gene inactivations as measured by real-time RT-PCR assays. (TIF) [file pgen.1002542.s004.tif]

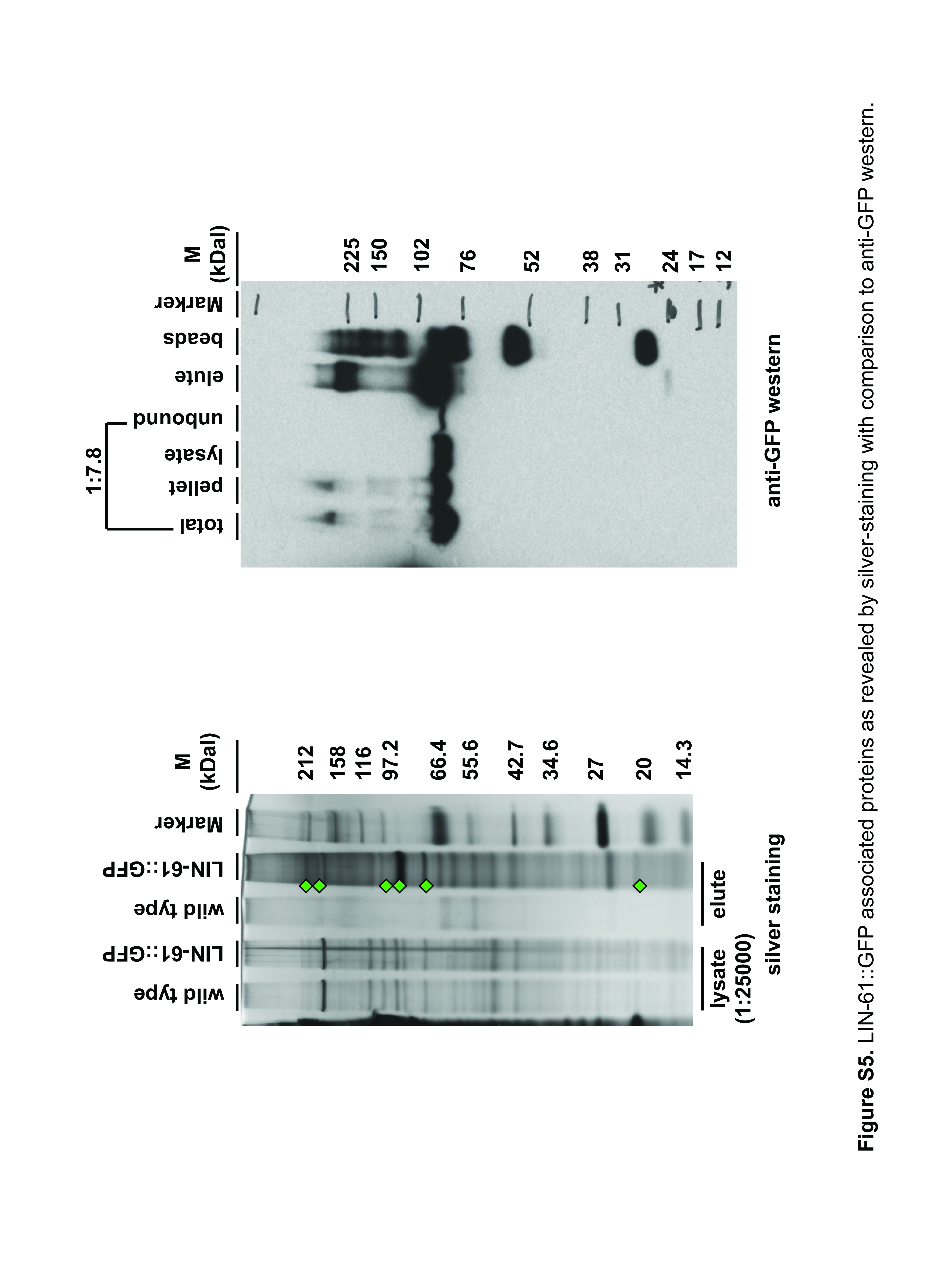

Supplement: Figure S5 — LIN-61::GFP associated proteins as revealed by silver-staining with comparison to anti-GFP western. (TIF) [file pgen.1002542.s005.tif]

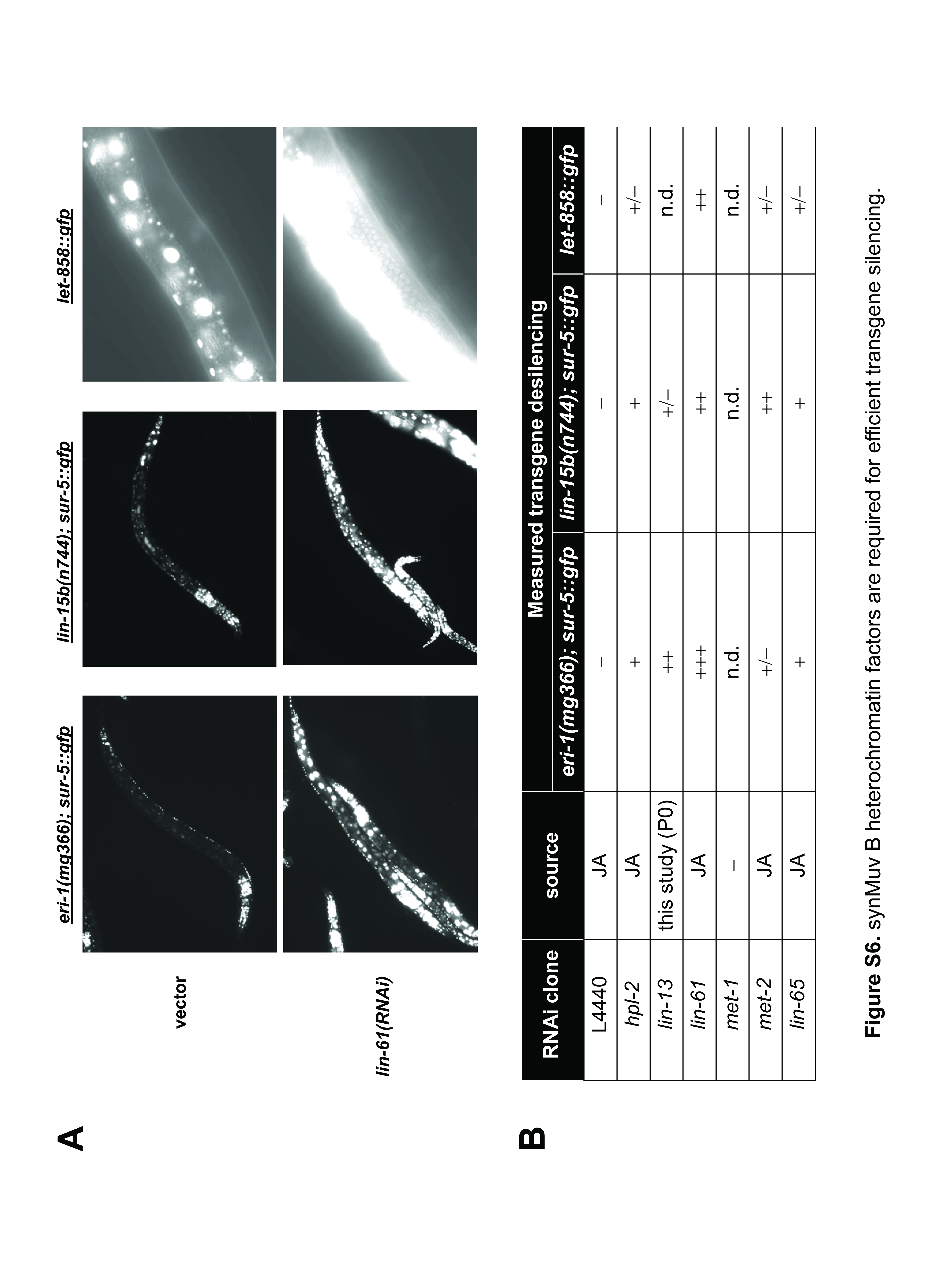

Supplement: Figure S6 — synMuv B heterochromatin class proteins are required for transgene silencing. (A) Fluorescent microscope images showing the desilencing effect of lin-61(RNAi) on different scenarios of transgene silencing (eri-1-induced, DRM-induced, and natural germline silencing). (B) Summary of transgene desilencing phenotypes upon RNAi inactivation of synMuv B heterochromatin class genes as measured by GFP fluorescence for transgene expression. −: no desilencing effect detected compared to vector RNAi control. +/− to +++: different levels of desilencing, ranging from marginal to strong. (TIF) [file pgen.1002542.s006.tif]

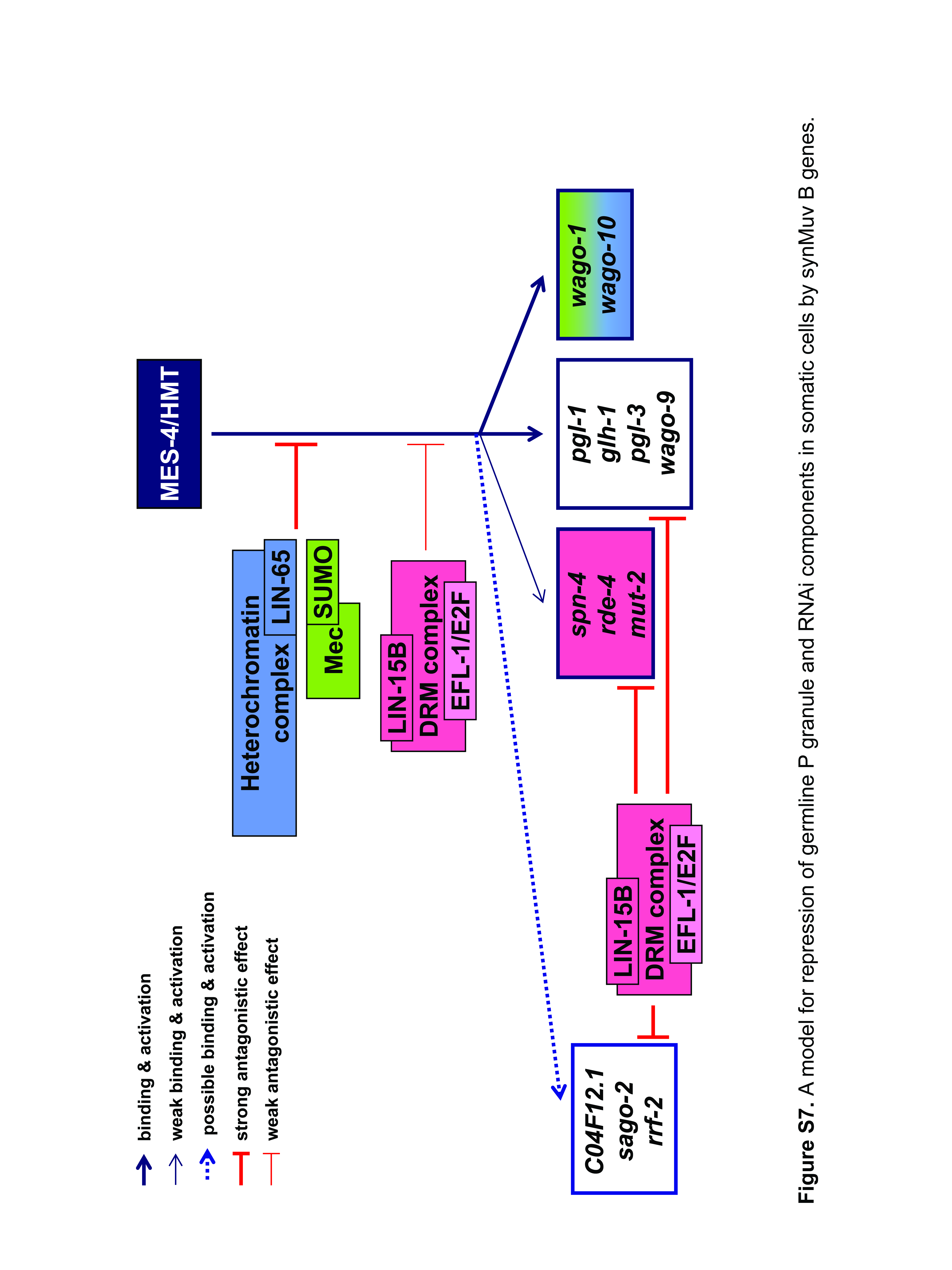

Supplement: Figure S7 — A model for repression of germline P granule and RNAi components in somatic cells by synMuv B genes. (TIF) [file pgen.1002542.s007.tif]
